# Three novel QTLs for FHB resistance identified and mapped in spring wheat PI672538 by bulked segregant analysis of the recombinant inbred line

**DOI:** 10.3389/fpls.2024.1409095

**Published:** 2024-07-29

**Authors:** Qianglan Huang, Xin Li, Qing Li, Shengfu Zhong, Xiuying Li, Jiezhi Yang, Feiquan Tan, Tianheng Ren, Zhi Li, Yang Suizhuang

**Affiliations:** ^1^ Wheat Research Institute, School of Life Sciences and Engineering, Southwest University of Science and Technology, Mianyang, Sichuan, China; ^2^ Provincial Key Laboratory of Plant Breeding and Genetics, Sichuan Agricultural University, Chengdu, Sichuan, China; ^3^ Department of Biology and Chemistry, Chongqing Industry and Trade Polytechnic, Fuling, Chongqing, China; ^4^ Wheat Research Institute, Neijiang Academy of Agricultural Sciences, Neijiang, Sichuan, China

**Keywords:** wheat, FHB, QTL mapping, BSA-seq, PI672538

## Abstract

**Introduction:**

*Fusarium* head blight (FHB) has a large influence on both the yield and quality of wheat grain worldwide. Host resistance is the most effective method for controlling FHB, but unfortunately, very few genetic resources on FHB resistance are available; therefore, identifying novel resistance genes or quantitative trait loci (QTLs) is valuable.

**Methods:**

Here, a recombinant inbred line (RIL) population containing 451 lines derived from the cross L661/PI672538 was sown in four different environments (2019CZ^a^, 2019CZ^b^, 2021QL and 2021WJ).

**Results:**

Five QTLs, consisting of two previously reported QTLs (*FhbL693a* and *FhbL693b*) and three new QTLs (*FhbL693c*, *FhbL693d* and *FhbL693e*), were identified. Further investigation revealed that *FhbL693b*, *FhbL693c* and *FhbL693d* could be detected in all four environments, and *FhbL693a* and FhbL693e were detected only in 2019CZb and 2021WJ, respectively. Among the QTLs, the greatest contribution (10.5%) to the phenotypic variation effect (PVE) was *FhbL693d* in 2021WJ, while the smallest (1.2%) was *FhbL693e* and *FhbL693a* in 2019CZ^b^. The selection of *5Dindel-4* for *FhbL693d*, *4Aindel-7* for *FhbL693c* and *3Bindel-24* for *FhbL693b* decreased the number of damaged spikelets by 2.1, and a new line resistant to FHB named H140-2 was developed by marker-assisted selection (MAS).

**Discussion:**

These results could help to further improve FHB resistance in the future.

## 1 Introduction

*Fusarium* head blight (FHB), which is caused mainly by *Fusarium graminearum* Schwabe, is a destructive wheat (*Triticum aestivum* L., 2n = 6X = 42, genome AABBDD) disease worldwide (Bai and Shaner, 2004; Huang and Luo, 2021). FHB not only causes heavy yield loss but also decreases grain quality and functionality when contaminated with mycotoxins, such as deoxynivalenol and nivalenol (Zwart et al., 2008; Sobrova et al., 2010). To date, no single strategy has been shown to be effective at alleviating the effects of FHB, but one promising avenue is the development of more resistant wheat cultivars to control this disease. At present, only few FHB-resistant varieties have already been released. Therefore, it is emergency to develop wheat cultivars resistant to FHB.

The main sources of FHB resistance utilized in current wheat breeding mainly involve *Fhb1* (Bai and Shaner, 2004; Buerstmayr et al., 2010; Li et al., 2019; Su et al., 2019) and *Fhb7* (Guo et al., 2015; Wang et al., 2020). There is a potential risk of resistance loss and disease epidemics if there are only a few resistance sources across large crop production areas. Therefore, discovering and identifying new genes conferring resistance to FHB are essential in wheat breeding. Genetics research has shown that resistance to FHB in wheat is a quantitative trait controlled by numerous quantitative trait loci (QTLs) and affected by environmental conditions. Over 100 QTLs have been reported to be associated with FHB resistance (Buerstmayr et al., 2010, 2020). Some of the FHB resistance-associated loci have been previously mapped and designated with a gene name: *Fhb1* (Cuthbert et al., 2006) and *Fhb2* (Cuthbert et al., 2007) were derived from *T. aestivum* cv. Sumai 3; *Fhb3* was derived from *Leymus racemosus* (Qi et al., 2008); *Fhb4* (Xue et al., 2010) and *Fhb5* (Xue et al., 2011) were derived from *T. aestivum* cv. Wangshuibai; *Fhb6* was derived from *Elymus tsukushiensis* (Liu et al., 2006); *Fhb7* was derived from *Thinopyrum ponticum* (Guo et al., 2015); and *Fhb8* was derived from Wangshuibai (Wang et al., 2023). In addition, few wheat varieties exhibit a high level of FHB resistance, and no source of immunity has been identified (Bai and Shaner, 2004; Yu et al., 2008; He et al., 2014). Therefore, identifying and mapping new resistance genes are important in wheat breeding programs.

Reduced-representation genome sequencing (RRGS) was previously developed, and its use has been widely accepted. Several methods were used into RRGS to create single-nucleotide polymorphisms (SNPs) and insertions/deletions (InDels) by bulked segregant analysis (BSA) (Song et al., 2017; Li et al., 2018; Wang et al., 2018; Ye et al., 2022; Zhang et al., 2022; Zhong et al., 2023). These methods include the restriction site-associated DNA (RAD) method (Davey and Blaxter, 2011), the genotyping-by-sequencing (GBS) method (Elshire et al., 2011), the 2b-RAD method (Wang et al., 2012), the double-digest RAD (ddRAD) method (Peterson et al., 2012), and the specific-length amplified fragment (SLAF) method (Zhu et al., 2015). These methods accelerated the speed of QTL mapping and gene cloning.

The wheat germplasm PI672538, derived from the wheat grass *Thinopyrum intermedium* (Host) (Barkworth and D. R. Dewey) (2n=6x=42; JJJ^s^J^s^SS) (syn. *Elytrigia intermedia* (Host) Nevski), is resistant to FHB (Liu et al., 2015). The highly resistant line PI672538 carried the FHB resistance genes *FhbL693a* and *FhbL693b*, which could explain only 20% of the phenotypic variation in FHB resistance in the F_2:3_ population (Li et al., 2017), which indicated the possibility that there could be other QTLs in PI672538. Moreover, the chromosomal regions of these genes were too large for map-based cloning. Therefore, identifying new QTLs and precisely mapping previously identified QTLs in PI672538 would be valuable.

The objectives of this study were (a) to identify all detectable FHB resistance QTLs in PI672538 using BSA sequencing (BSA-seq), (b) to precisely map them using newly developed molecular markers and (c) to select new lines with strong FHB resistance by molecular marker-assisted selection (MAS). The results of this study could be valuable for improving FHB resistance in wheat.

## 2 Materials and methods

### 2.1 Plant materials and population construction

PI672538 is resistant to FHB (Liu et al., 2015; Li et al., 2017; Huang and Luo, 2021), while L661 is susceptible to FHB. PI672538 and L661 were sister lines, and they were both derived from the wheat grass *Thinopyrum intermedium* (Liu et al., 2015). A total of 337 F_2:7_ plants derived from the cross L661/PI672538 were identified to construct the FHB resistance pool (R pool) and susceptible pool (S pool). Twenty-four resistant and 20 susceptible lines were selected as the R pool and S pool, respectively, for BSA-seq. To further accurate mapping FHB QTLs, we reconstructed 451 F_10_ RILs derived from F_2:7_ plants of the same cross, L661/PI672538. Of the 451 F_10_ RILs, 192 were selected for linkage analysis.

### 2.2 Evaluation of the reaction to FHB

The parental lines L661 and PI672538 and 337 F_2:7_ plants were grown by row (30 cm row space distance and 1.5 m length) in Wenjiang (lat. 30°43’ N, long. 103°52’ E) in 2014-2015; in Wenjiang in 2015-2016; and in Wenjiang, Neijiang (lat. 29°31’N, long. 104°56’ E) and Fuling (lat. 29°38’ N, long. 107°22’ E) in 2016-2017. To evaluate FHB resistance in field trials from 2014–2017, more than 10 randomly selected spikes from each treatment for each genotype were inoculated with *F. graminearum* No F_15_, which was provided by Professor Gong Guoshu, Plant Pathology Laboratory, Sichuan Agricultural University. *F. graminearum* No F_15_ was used as an inoculum, and conidia were prepared according to previous methods (Huang and Luo, 2021). At early anthesis, 10 µl of conidial suspension (~1000 conidia/spikelet) was injected into two small opposite-direction flowers in the central spikelet of a spike using a syringe (Hamilton, Reno, NV, USA). The inoculated spikes were then covered with plastic bags to maintain a relatively high humidity, and the plastic bags were removed at 72 h after inoculation. The number of diseased spikelets (NDS) caused by *Fusarium*, which is associated with the deoxynivalenol content, was used to evaluate FHB resistance. The NDS at 21 days after inoculation (DAI) was recorded, and the average of all the inoculated spikes from the same treatment was used to represent the value of the treatment.

The parental lines L661 and PI672538 and 451 F_10_ RILs from the cross L661/PI672538 were grown individually spaced in Chongzhou (lat. 30°54’N, long. 103°65’E) twice in 2018 and 2019 (identified as 2019CZ^a^ and 2019CZ^b^, respectively) and in Wenjiang and Qionglai (lat. 30°42’N, long. 103°47’E) from 2020–2021 (identified as 2021WJ and 2021QL, respectively). The resistance to FHB of 451 F_10_ RILs was evaluated in field trials by the same method described above.

### 2.3 DNA isolation

For BSA-seq, DNA from the parents and two bulk plant samples were extracted from fresh leaves using a DNA Secure Plant Kit (Shenggong, Chengdu). For linkage analysis and QTL mapping, genomic DNA was isolated using the modified cetyltrimethylammonium bromide (CTAB) method. In the modified CTAB method, the 0.6 times volume isopropyl alcohol with -20°C were used to precipitate DNA in 3 min and immediately mixed for centrifuging. The DNA was measured using a DNA Nano 2000, and the concentration was adjusted to 150 ng/µl.

### 2.4 Generation and analysis of BSA-seq data

The DNA from the R and S pools was extracted using the CTAB method (Zhong et al., 2023); the R and S pools were constructed by mixing the same amounts of fresh leaves from 24 R plants and 20 S plants to perform BSA. RRGS was executed according to the ddRAD protocol (Peterson et al., 2012) and using the Illumina HiSeq 2500 platform (Illumina, Inc. 9885 Towne Centre Drive, San Diego, CA, USA) by Majorbio (Shanghai, China). In brief, two restriction enzymes, TaqI and MseI, were used to digest the DNA of the R/S pools and parental lines. Then, restriction fragments were purified and separated via electrophoresis on a 2% (w/v) agarose gel. Approximately 380 bp DNA fragments were used to construct a paired-end sequencing library for further sequencing to yield 2 × 150 bp paired-end reads. Raw paired-end reads were generated using the standard procedure of Illumina base calling.

Clean reads were obtained and checked by removing both adaptor and poor-quality reads (length less than 20 bp or more than 10% N bases) and by eliminating short reads (length less than 25 bp) using FASTX-Toolkit (v 0.0.13) (Gordon, Cold Spring Harbor, NY, USA) and FastQC. The clean reads were subsequently aligned to the wheat IWGSC v1.0 reference genome (International Wheat Genome Sequencing Consortium, 2018) using HISAT2 with default parameter values (Kim et al., 2015), and the alignment files were subsequently converted into BAM files using SAMtools (Li et al., 2009). Finally, variations such as SNPs and InDels were identified by integrating analyses using SAMtools, BEDTools (Quinlan and Hall, 2010) and GATK (McCormick et al., 2015). SNPs and InDels were defined on the basis of a Fisher’s exact test score > 30, a Qual by Depth (QD) value < 2, or coverage in the bulk pool and parents < 10× and 5×, respectively. In addition, only the SNP and InDel markers at completely corresponding loci between the two bulk pools and two parents were used for QTL mapping. The average SNP index and ΔSNP index were used to physically map the QTLs, and the values were calculated via the three methods described below.

First, we conducted BSA of variants (SNPs and InDels) between the R pool and S pool and the parents using the sliding window algorithm (window = 2 Mb and step=10 kb).

Second, we conducted BSA of variants (SNPs and InDels) between the R pool and S pool without parents using the sliding window algorithm (window = 2 Mb and step=10 kb). The SNP index or InDel index and the ΔSNP or ΔInDel index were calculated for all physical positions to identify candidate regions associated with the FHB trait. The SNP index and InDel index were calculated by totaling the number of reads harboring an SNP compared to the reference genome sequence and dividing this total by the total number of reads. The ΔSNP or ΔInDel indices were calculated by subtracting the SNP index/InDel index of the S pool from that of the R pool.

Third, to further develop a molecular marker for the FHB trait, the variation indices were filtered by a wheat genome annotation information file (iwgsc_refseqv1.0_HighConf_2017Mar13.gff3) to obtain a high-confidence variation index because the genome annotation is highly specific and contains almost all of the whole-genome exome (https://wheat-urgi.versailles.inra.fr/Projects/IWGSC). We conducted BSA of variants (SNPs and InDels) between the R pool and S pool without parents using a sliding window algorithm (window = 50 Mb and step=50 Mb). The SNP index or InDel index and ΔSNP or ΔInDel index were calculated using the same methods above. A circle map was generated to show the BSA results using TBtools v0.6673 software (Chen et al., 2020).

### 2.5 Molecular marker development

To confirm the BSA-seq results, simple sequence repeat (SSR) and InDel markers were designed and developed using the NCBI online tool Primer-BLAST (https://www.ncbi.nlm.nih.gov/tools/primer-blast/). SSR markers were designed near or containing the SNP or InDel loci associated with FHB according to the BSA results. InDel markers were designed according to the conserved sequences containing the InDel loci for InDels longer than 5 bp according to BSA-seq. The InDel markers were first used for screening polymorphisms between the two parents and between the two pools. The polymorphic InDel markers were utilized in linkage analysis of 192 F_10_ RILs, and the linked markers were subsequently used for QTL mapping in the 451 F_10_ RIL plants. PCR amplification was performed in a 15 μl reaction mixture containing 150 ng of template DNA, 60 μmol of F/R primers, and 7.5 µl of 2x T5 Supper PCR Mix (PAGE) provided by Tsingke Biology Company. All PCR conditions were set as follows: initial denaturation at 98°C for 2-3 min; 35 cycles of 98°C for 15 s, 56°C for 15 s, and 72°C for 15 s; and a final extension at 72°C for 2 min. The PCR products were separated by 8% nondenaturing PAGE and visualized by silver staining.

### 2.6 Linkage analysis and QTL mapping

Linkage analysis of polymorphic molecules was performed for 192 RILs by an independent sample t test using IBM SPSS Statistics 19 software (SPSS, Inc., Chicago, IL). Linked markers were subsequently used to construct a genetic linkage map of 451 F_10_ RILs via the Map function in QTL IciMapping 4.0 software. In the Map function, the parameters were set as follows: the markers were grouped by logarithm of odds (LOD)=7 using the nnTwoOpt algorithm, the SARF rule, and a window size=5 using Kosambi’s mapping function. After the Map function was completed, an input file was obtained, which was used for the BIP function after the corresponding FHB phenotypic data were added. In the BIP function, the null phenotype was replaced by the mean, step distance was 1 cM, PIN was 0.001, and the mapping method was ICIM-ADD. The LOD was determined by a 1000 permutation test, and type I error was set as 0.05.

### 2.7 QTL effect analysis and FHB-resistant line selection

Stable and reliable QTLs were selected according to their QTL detection efficiency and phenotypic variation effect (PVE) in this study. The linked QTL markers were selected according to a close genetic distance to the QTL location. To determine the effect of QTLs on FHB spread resistance, we calculated the NDS mean under linked marker-assisted selection conditions. To screen the FHB-resistant lines, linked markers were used for MAS combined with FHB resistance evaluation under field conditions.

## 3 Results

### 3.1 FHB reaction in the F_2:7_ and F_10_ RIL populations

**Figure 1:**
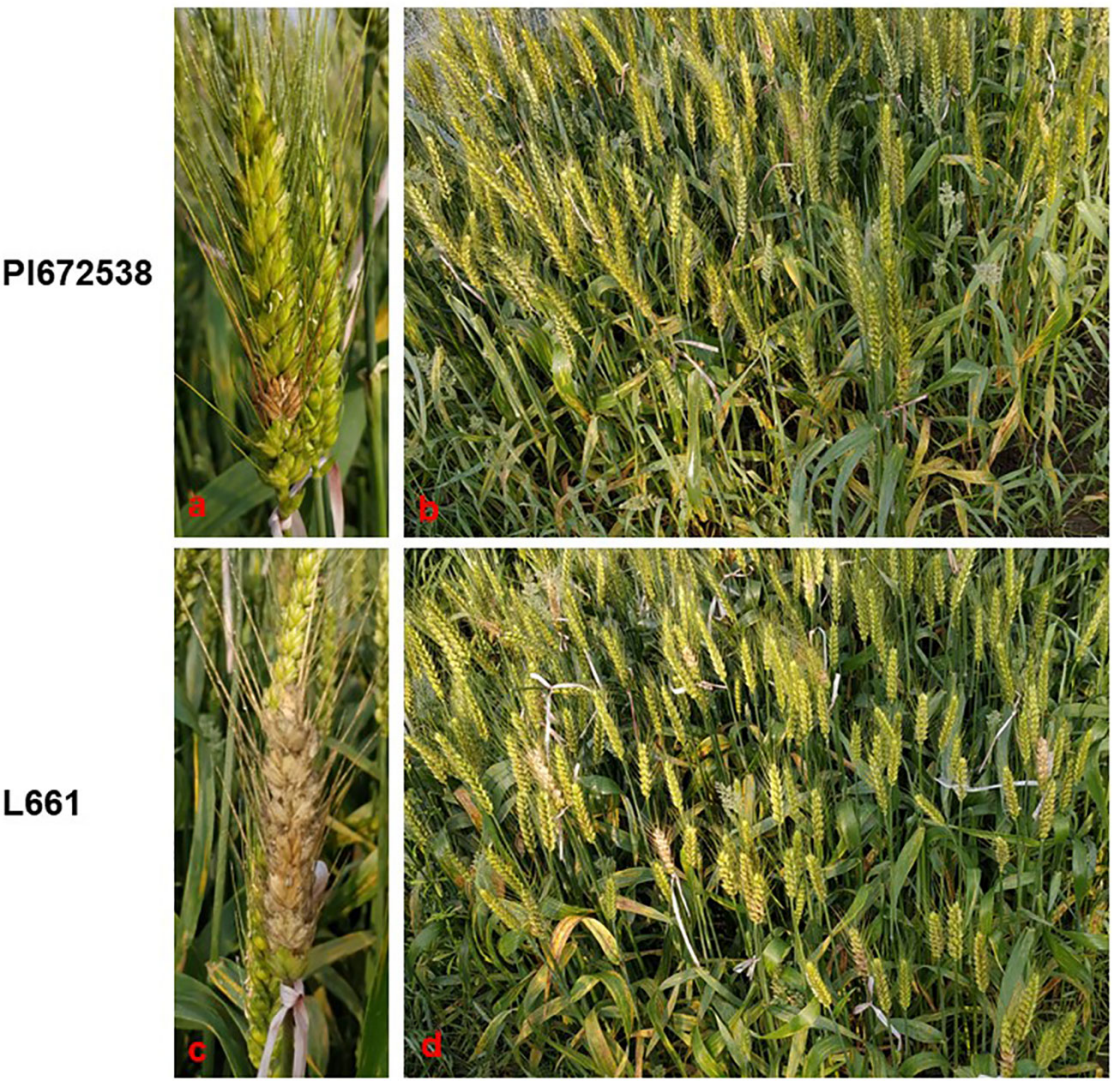
The FHB resistance performance of PI672538 and L661 at 21 days after *Fusarium* inoculation in field in 2019CZ^a^. **(A–D)** respectively represent the FHB resistance performance of spike of PI672638, population of PI672538, spike of L661, and population of L661.

A four-year field test showed that PI672538 was resistant to FHB, while L661 was susceptible to FHB (**Table 1**; **Figure 1**). The NDS per spike of F_2:7_ and F_10_ plants exhibited continuous variation to some degree. Correlation analysis revealed that the NDS of the F_2:7_ RIL population during 2015–2017 exhibited a weak correlation (**Supplementary Table 1**), while the NDS of the F_10_ RIL population during 2019–2021 exhibited a stronger correlation (R^2 ^= 0.272–0.521, P<0.01) (**Table 2**), illustrating that the F_10_ RIL population could be used for QTL mapping of FHB. The standard errors of NDS_QL2021, NDS_WJ2021, NDS_CZ2019 and NDS_CZCF2019 exhibited a nearly orthotropic distribution (**Table 3**), which is suitable for QTL mapping.

**Table 1:** The FHB resistance evaluation during 2015-2021 year.

Genotype	2015WJ		2017NJ		2017WJ		2019CZ		2021WJ		2021QL	
	N	NDS	N	NDS	N	NDS	N	NDS	N	NDS	N	NDS
L661	18	8.6 ± 1.5 a	30	5.9 ± 0.8 a	29	5.2 ± 0.6 a	19	8.2 ± 1.1 a	24	5.1 ± 0.6 a	20	4.2 ± 0.4 a
PI672538	21	5.9 ± 1.0 b	27	2.6 ± 0.3 b	30	2.7 ± 0.2 b	23	2.3 ± 0.7 b	24	2.2 ± 0.1 b	26	2.2 ± 0.1 b

N, the number of inoculated spikes; NDS, the number of diseased spikelets per spike. The means in a column followed by the same lowercase letter(s) are not significantly different at the 5% probability level in the same year.

**Table 2:** Person correlation analysis of the number of diseased spikelets (NDS) in 451 F_10_ RILs.

	NDS_QL2021	NDS_WJ2021	NDS_CZ2019	NDS_CZCF2019
NDS_QL2021	1			
NDS_WJ2021	0.399^**^	1		
NDS_CZ2019	0.272^**^	0.462^**^	1	
NDS_CZCF2019	0.344^**^	0.521^**^	0.515^**^	1

NDS_QL2021, the number of diseased spikelets in Qionglai in 2021; NDS_WJ2021, the number of diseased spikelets in Wenjiang in 2021; NDS_CZ2019, the number of diseased spikelets in Chongzhou in 2019; and NDS_CZCF2019, the number of diseased spikelets in Chongzhou replicate in 2019. ^**, ^The correlation coefficient is significant at the P<0.01 level.

**Table 3:** Standard error of the mean of the number of diseased spikelets (NDS) per spike in the F_10_ RIL population and the test of normal distribution.

SE of NDS	N	Mean	Skewness	Kurtosis
NDS_CZ2019	448	3.59 ± 0.09	0.01 ± 0.12	-1.03 ± 0.23
NDS_CZCF2019	249	1.85 ± 0.07	0.96 ± 0.15	0.55 ± 0.31
NDS_QL2021	448	0.36 ± 0.01	-0.09 ± 0.12	0.15 ± 0.23
NDS_WJ2021	445	0.17 ± 0.01	0.94 ± 0.12	1.20 ± 0.23

SE of NDS, standard error of the mean of the number of diseased spikelets (NDS); N, population size; NDS_QL2021, the number of diseased spikelets in Qionglai in 2021; NDS_WJ2021, the number of diseased spikelets in Wenjiang in 2021; NDS_CZ2019, the number of diseased spikelets in Chongzhou in 2019; NDS_CZCF2019, the number of diseased spikelets in Chongzhou replicate in 2019.

### 3.2 Assessment of FHB in the R and S pools

To map FHB QTLs, 24 FHB-resistant lines and 20 FHB-susceptible lines were selected from the F_2:7_ population as the resistant and susceptible pools, respectively. In addition, the NDS of the F_2:7_ population in 2017 and 2018 in Wenjiang in the R pool and S pool showed that FHB resistance was stable and accurate (**Supplementary Table 2**), which illustrated that the R pool and S pool could be used for BSA-seq.

### 3.3 BSA analysis, marker design and linkage analysis

After applying BSA-seq, the resistant and susceptible pools produced 49.43 and 33.15 Gb of data, respectively. After quality control, <1% of the raw read pairs were filtered. Trimmed reads were aligned to the wheat IWGSC v1,0 reference genome. In total, 85.04% and 85.81% of the filtered read pairs were properly mapped in the R pool and S pool, respectively (**Table 4**). Subsequent SNP calling identified 2180669 (108432) high-quality variants (SNPs and InDels) between the R pool and S pool. Then three methods were taken to analyze the variants of BSA.

**Table 4:** Sequence and genome coverage depth data.

Sample ID	Mapped Ratio (%)	Properly Mapped (%)	Insert Size (bp)	Real Depth	Genome Coverage (1X) (%)	Genome Coverage (5X) (%)
PI672538	99.45	77.47	531.3	8.26	31.64	9.04
L661	99.89	84.76	495.4	10.93	30.87	8.98
R pool	99.27	85.04	385.7	9.49	35.56	13.39
S pool	99.67	85.81	390.0	7.81	29.08	9.21

**Figure 2:**
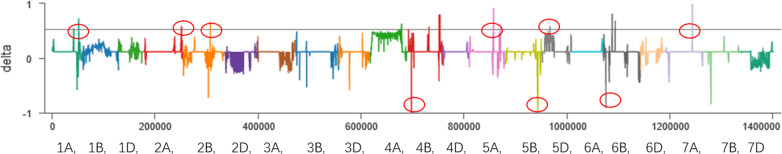
BSA results of the sliding window algorithm (window = 2 Mb, step= 10 kb). X-axis, the chromosome and position; index1, the SNP index in the resistant pool; index2, the SNP index in the susceptible pool; delta, Δ(SNP index).

First, the BSA-seq results between R pool and S pool and parents showed that some regions on chromosomes 1A, 2A, 2B, 4B, 5A, 5B, 5D, 6B and 7A may be associated with FHB traits (**Figure 2**). Polymorphic and linkage analysis of the pooled and 192 RIL populations showed that no marker was linked in 192 RIL populations.

**Figure 3:**
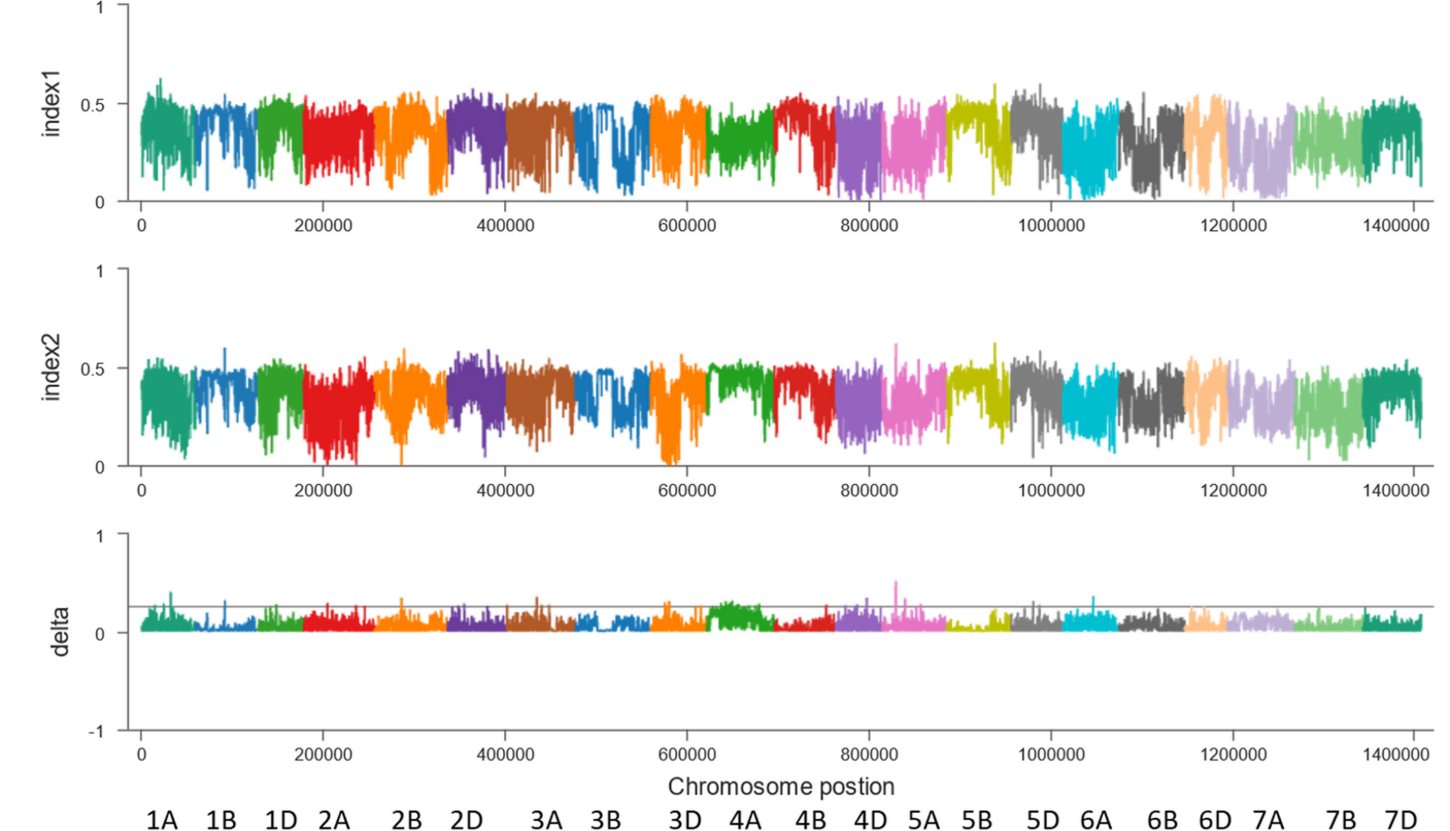
BSA results for the 2M10K sliding window without parents. X-axis, the chromosome and position; index1, the SNP index in the resistant pool; index2, the SNP index in the susceptible pool; delta, Δ(SNP index).

Second, the BSA-seq results between the R pool and S pool without parents showed that some regions on chromosomes 1A, 1B, 1D, 2A, 2B, 2D, 3A, 3D, 4A, 4D, 5A, 5D and 6A may be associated with FHB (**Figure 3**). The sequences containing or near these regions were extracted for designing markers. Polymorphic and linkage analysis of the pooled and 192 RIL populations showed that markers on chromosomes 2B, 4A and 5D were linked in the 192 F_10_ RIL populations (**Table 5**).

**Table 5:** Independent sample t tests of partial InDel and SSR markers in the NDS of 192 RILs.

Marker	Position	NDS_CZ2019		NDS_CZCF2019		NDS_WJ2021		NDS_QL2021	
		A	B	A	B	A	B	A	B
*1Bindel-1*	121730466	6.0 ± 0.3	5.9 ± 0.3	4.0 ± 0.2*	3.3 ± 0.2	2.3 ± 0.0	2.3 ± 0.0	3.3 ± 0.1	3.2 ± 0.1
*1Bindel-2*	142612623	4.7 ± 0.5*	6.2 ± 0.4	3.0 ± 0.2	3.6 ± 0.2	2.2 ± 0.0	2.3 ± 0.0	3.0 ± 0.1	3.2 ± 0.1
*1Bindel-4*	374519769	5.9 ± 0.3	6.2 ± 0.3	3.9 ± 0.2	3.7 ± 0.2	2.3 ± 0.0	2.3 ± 0.0	3.3 ± 0.1	3.3 ± 0.1
*1Bindel-6*	532662731	5.7 ± 0.3	5.8 ± 0.3	3.6 ± 0.2	3.6 ± 0.2	2.2 ± 0.0	2.3 ± 0.0	3.2 ± 0.1	3.2 ± 0.1
*2Bindel-1*	44583841	6.1 ± 0.4	5.9 ± 0.2	4 ± 0.3	3.6 ± 0.2	2.3 ± 0.1	2.2 ± 0.0	3.3 ± 0.1	3.2 ± 0.1
*2Bindel-4*	585884064	6.5 ± 0.3*	5.5 ± 0.3	4.1 ± 0.2*	3.4 ± 0.2	2.3 ± 0.0	2.3 ± 0.0	3.3 ± 0.1	3.2 ± 0.1
*Xmag3930B-2B*	2B	5.9 ± 0.3	6.2 ± 0.3	3.6 ± 0.2	3.9 ± 0.2	2.3 ± 0.0	2.3 ± 0.0	3.3 ± 0.1	3.3 ± 0.1
*Xgwm410-2B*	2B	6.1 ± 0.3	6.2 ± 0.3	3.9 ± 0.2	3.7 ± 0.2	2.3 ± 0.0	2.3 ± 0.0	3.2 ± 0.1	3.4 ± 0.1
*Xbarc1064-2B*	2B	6.4 ± 0.3	5.6 ± 0.3	4.0 ± 0.2*	3.5 ± 0.2	2.3 ± 0.0	2.3 ± 0.0	3.4 ± 0.1	3.3 ± 0.1
*Xcn16-2B*	2B	6.7 ± 3.6*	5.8 ± 0.2	4.4 ± 0.3**	3.4 ± 0.1	2.3 ± 0.0	2.2 ± 0.0	3.4 ± 0.1	3.2 ± 0.1
*Xgwm148-2B*	2B	6.1 ± 0.3	6.2 ± 0.3	3.7 ± 0.2	3.9 ± 0.2	2.3 ± 0.0	2.3 ± 0.0	3.4 ± 0.1	3.2 ± 0.1
*3Bindel-2*	29852604	5.5 ± 0.3*	6.5 ± 0.3	3.5 ± 0.2*	4.0 ± 0.2	2.3 ± 0.0	2.3 ± 0.0	3.2 ± 0.1*	3.4 ± 0.1
*3Bindel-4*	68390538	6 ± 0.4	5.2 ± 0.3	3.7 ± 0.3	3.2 ± 0.2	2.3 ± 0.1	2.2 ± 0.0	3.3 ± 0.1	3.0 ± 0.1
*3Bindel-9*	320622057	5.2 ± 0.3**	6.9 ± 0.3	3.3 ± 0.2**	4.4 ± 0.2	2.2 ± 0.0*	2.3 ± 0.0	3.0 ± 0.1**	3.6 ± 0.1
*3Dindel-1*	38551060	5.9 ± 0.3	6.1 ± 0.3	3.6 ± 0.2	3.9 ± 0.2	2.3 ± 0.0	2.3 ± 0.0	3.2 ± 0.1	3.3 ± 0.1
*3Dindel-2*	38949397	5.8 ± 0.3	5.9 ± 0.4	3.7 ± 0.2	3.6 ± 0.3	2.2 ± 0.0	2.3 ± 0.0	3.1 ± 0.1	3.3 ± 0.1
*4Aindel-1*	27670704	6.6 ± 0.3**	4.9 ± 0.3	4 ± 0.2*	3.3 ± 0.2	2.3 ± 0.0**	2.1 ± 0.0	3.5 ± 0.1**	3.0 ± 0.1
*4Aindel-3*	58532210	6.6 ± 0.2**	4.6 ± 0.3	4.1 ± 0.2**	3.0 ± 0.1	2.3 ± 0.0**	2.1 + 0.0	3.4 ± 0.1**	2.9 ± 0.1
*4Aindel-4*	65633552	6.5 ± 0.2**	4.7 ± 0.3	4 ± 0.2**	3.0 ± 0.2	2.3 ± 0.0**	2.1 + 0.0	3.4 ± 0.1**	2.9 ± 0.1
*4Aindel-6*	180729886	6.4 ± 0.2**	4.6 ± 0.3	4 ± 0.2**	3.0 ± 0.2	2.3 ± 0.0**	2.1 + 0.0	3.4 ± 0.1**	2.8 ± 0.1
*4Aindel-7*	236872635	6.4 ± 0.2**	4.6 ± 0.3	4 ± 0.3**	2.9 ± 0.1	2.3 ± 0.0**	2.1 + 0.0	3.4 ± 0.1**	2.8 ± 0.1
*5Bindel-1*	664470704	6.7 ± 0.3**	5.3 ± 0.2	4.0 ± 0.2*	3.3 ± 0.2	2.3 ± 0.0**	2.2 ± 0.0	3.5 ± 0.1**	3.1 ± 0.1
*5Dindel-2*	47080989	6.8 ± 0.3**	5.1 ± 0.3	4.2 ± 0.2**	3.2 ± 0.2	2.3 ± 0.0**	2.2 ± 0.0	3.4 ± 0.1**	3.1 ± 0.1
*5Dindel-4*	153706503	7.1 ± 0.3**	4.9 ± 0.2	4.3 ± 0.2**	3.2 ± 0.1	2.4 ± 0.0**	2.2 ± 0.0	3.5 ± 0.1**	3.1 ± 0.1
*6Bindel-8*	689034083	5.9 ± 0.9	6.0 ± 0.8	3.3 ± 0.4	3.0 ± 0.3	2.2 ± 0.1	2.2 ± 0.1	3.1 ± 0.2	3.1 ± 0.2
*ms-11*	3B	6.3 ± 0.3	5.8 ± 0.3	4.1 ± 0.3	3.7 ± 0.2	2.3 ± 0.0	2.3 ± 0.0	3.5 ± 0.1*	3.2 ± 0.1
*ms5A-13*	607811064	6.2 ± 0.3	5.8 ± 0.3	3.7 ± 0.2	3.6 ± 0.2	2.3 ± 0.0	2.2 ± 0.0	3.3 ± 0.1	3.3 ± 0.1
*ms5A-3*	470150021	6.3 ± 0.3	5.7 ± 0.3	3.8 ± 0.2	3.6 ± 0.2	2.3 ± 0.0	2.2 ± 0.0	3.3 ± 0.1	3.3 ± 0.1
*ms7A-7*	–	6.4 ± 0.3**	4.7 ± 0.3	4.1 ± 0.2**	2.9 ± 0.1	2.3 ± 0.0**	2.1 ± 0.0	3.4 ± 0.1**	2.8 ± 0.1
*wmc102.1*	–	6.5 ± 0.3*	5.5 ± 0.3	4.0 ± 0.2*	3.4 ± 0.2	2.3 ± 0.0*	2.2 ± 0.0	3.4 ± 0.1**	3.1 ± 0.1
*wmc231*	3B	5.3 ± 0.3**	6.6 ± 0.3	3.4 ± 0.2**	4.2 ± 0.2	2.2 ± 0.0**	2.3 ± 0.0	3.0 ± 0.1**	3.5 ± 0.1
*wmc533c*	3B	5.8 ± 0.3	4.6 ± 0.5	3.7 ± 0.2	2.8 ± 0.2	2.2 ± 0.0	2.2 ± 0.0	3.2 ± 0.1	2.9 ± 0.1
*5Dindel-10*	79220144	6.5 ± 0.3**	5 ± 0.3	4.0 ± 0.2**	3.2 ± 0.2	2.3 ± 0.0**	2.1 ± 0.0	3.4 ± 0.1**	3.0 ± 0.1
*5Dindel-6*	53842984	5.7 ± 0.3	6.3 ± 0.3	3.6 ± 0.2	3.6 ± 0.2	2.3 ± 0.0	2.3 ± 0.0	3.2 ± 0.1	3.3 ± 0.1
*3Bindel-14*	258355404	5.2 ± 0.3**	6.8 ± 0.3	3.2 ± 0.2**	4.3 ± 0.2	2.2 ± 0.0**	2.3 ± 0.0	2.9 ± 0.1**	3.6 ± 0.1
*3Bindel-22*	320881284	5 ± 0.3**	6.6 ± 0.3	3.0 ± 0.2**	4.2 ± 0.2	2.2 ± 0.0*	2.3 ± 0.0	2.9 ± 0.1**	3.5 ± 0.1
*3Bindel-24*	328489242	5.1 ± 0.3**	7 ± 0.3	3.2 ± 0.2**	4.3 ± 0.2	2.2 ± 0.0**	2.4 ± 0.0	2.9 ± 0.1**	3.6 ± 0.1
*3Bindel-25*	331950894	5.1 ± 0.3**	7 ± 0.3	3.2 ± 0.2**	4.3 ± 0.2	2.2 ± 0.0**	2.3 ± 0.0	3.0 ± 0.1**	3.6 ± 0.1
*3Bindel-34*	274621461	5.3 ± 0.3**	7 ± 0.3	3.4 ± 0.2**	4.3 ± 0.2	2.2 ± 0.0**	2.4 ± 0.0	3.0 ± 0.1**	3.6 ± 0.1
*3Bindel-36*	282358935	5.2 ± 0.3**	7 ± 0.3	3.4 ± 0.2**	4.3 ± 0.2	2.2 ± 0.0**	2.4 ± 0.0	3.0 ± 0.1**	3.6 ± 0.1
*3Bindel-42*	302784826	5.1 ± 0.3**	7.1 ± 0.3	3.3 ± 0.2**	4.3 ± 0.2	2.2 ± 0.0**	2.4 ± 0.0	3.0 ± 0.1**	3.6 ± 0.1
*3Bindel-43*	305658046	5.1 ± 0.3**	7 ± 0.3	3.3 ± 0.2**	4.3 ± 0.2	2.2 ± 0.0**	2.4 ± 0.0	2.9 ± 0.1**	3.6 ± 0.1
*3Bindel-45*	308250513	4.8 ± 0.4**	7.4 ± 0.8	3.2 ± 0.2	4.0 ± 0.5	2.2 ± 0.3	2.3 ± 0.4	3.0 ± 0.1*	3.5 ± 0.2
*3Bindel-46*	311072382	5.3 ± 0.3**	7 ± 0.3	3.4 ± 0.2**	4.3 ± 0.2	2.2 ± 0.0**	2.4 ± 0.0	3.0 ± 0.1**	3.6 ± 0.1
*3Bindel-49*	325099648	5.2 ± 0.3**	7 ± 0.3	3.3 ± 0.2**	4.3 ± 0.2	2.2 ± 0.0**	2.4 ± 0.0	3.0 ± 0.1**	3.6 ± 0.1
*3Bindel-50*	325684304	5.4 ± 0.3**	7 ± 0.3	3.4 ± 0.2**	4.2 ± 0.2	2.2 ± 0.0**	2.4 ± 0.0	3.0 ± 0.1**	3.6 ± 0.1
*3Bindel-52*	328663127	5.2 ± 0.7	6.5 ± 0.9	2.6 ± 0.2*	3.7 ± 0.4	2.1 ± 0.0	2.3 ± 0.1	2.9 ± 0.2	3.3 ± 0.2
*3Bindel-53*	329472285	5.2 ± 0.3**	7 ± 0.3	3.3 ± 0.2**	4.3 ± 0.2	2.2 ± 0.0**	2.4 ± 0.0	3.0 ± 0.1**	3.6 ± 0.1
*3Bindel-59*	368096132	5.2 ± 0.3**	6.9 ± 0.3	3.3 ± 0.2**	4.4 ± 0.2	2.2 ± 0.0**	2.3 ± 0.0	3.0 ± 0.1**	3.6 ± 0.1
*3Bindel-60*	368845559	5.2 ± 0.3**	7 ± 0.3	3.3 ± 0.2**	4.3 ± 0.2	2.2 ± 0.0**	2.3 ± 0.0	3.0 ± 0.1**	3.6 ± 0.1

NDS_CZ2019, the number of diseased spikelets in Chongzhou in 2019; NDS_CZCF2019, the number of diseased spikelets in Chongzhou replicate in 2019; NDS_WJ2021, the number of diseased spikelets in Wenjiang in 2021; NDS_QL2021, the number of diseased spikelets in Qionglai in 2021; A, same genotype as L661; B, same genotype as PI672538. *, ** significant difference between the A genotype and B genotype at P < 0.05 and 0.01, respectively, in the same year/place. The gray background indicates markers associated with FHB.

**Figure 4:**
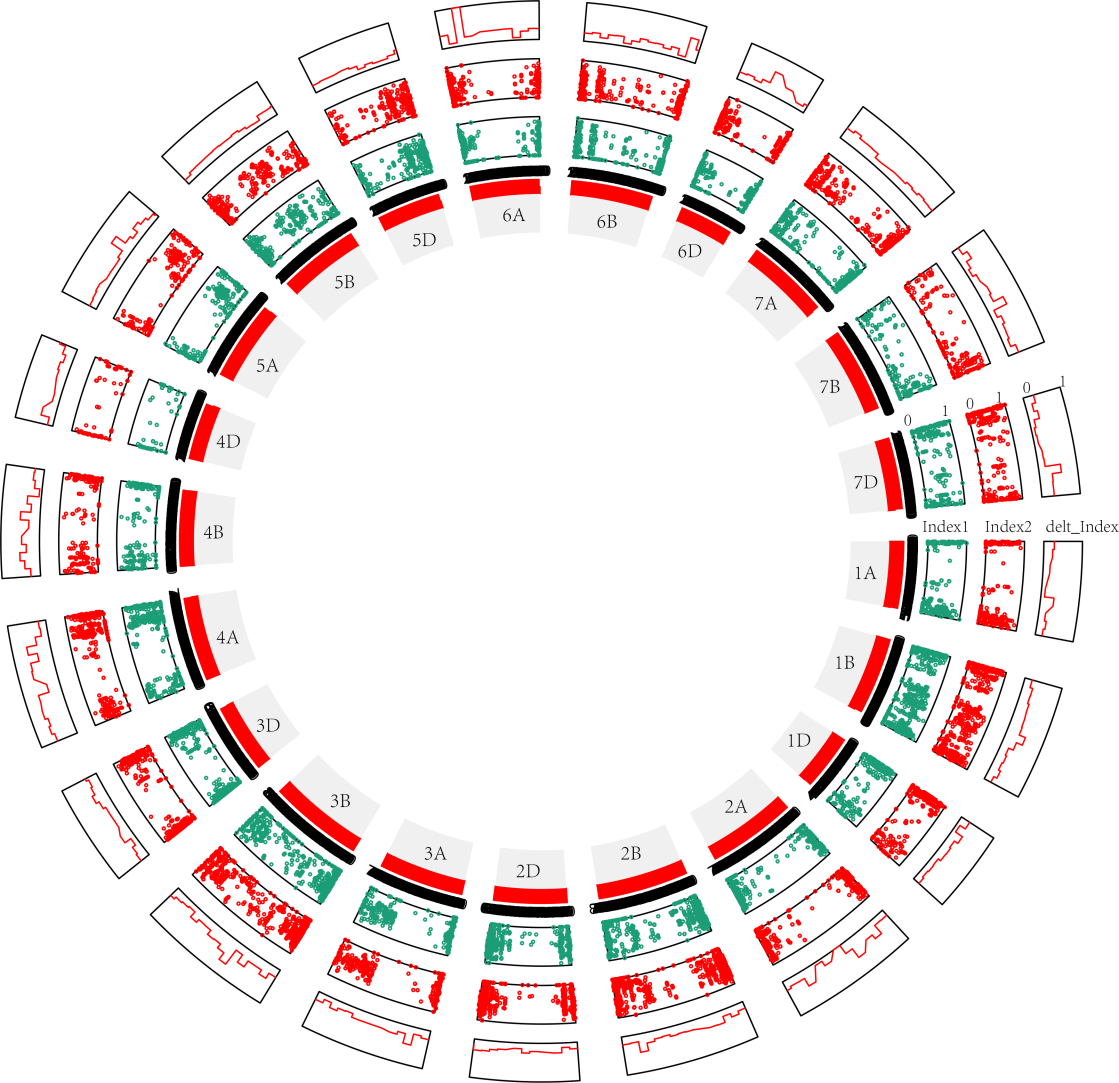
Modified BSA results for the 50M50M (window = 50 Mb, step= 50 Mb) sliding window without parents. The distribution of the variants is shown clockwise on the chromosome, and each window is 50 Mb in length. Index1, variants (SNPs and InDels) of S pool as dark blue points; Index2, variants (SNPs and InDels) of R pool as red points; delt_Index, △(SNPs and InDels) as red line. 1A, 1B, 1D … 7D are the chromosome numbers. The greater the delt_Index value, the greater the probability that QTL exists in a chromosome. The more obvious the crest, the greater the probability of QTL.

Third, in the modified BSA-seq results showed that regions on chromosomes 1B, 2B, 3B, 4A, 4B, 6A and 7D may be associated with FHB (**Figure 4**). The sequence was extracted to design markers. Polymorphic and linkage analysis of the pooled and 192 RIL populations showed that markers on chromosomes 1B, 2B, 3B and 4A were linked in the 192 RIL populations (**Table 5**).

### 3.4 QTL mapping

**Figure 5:**
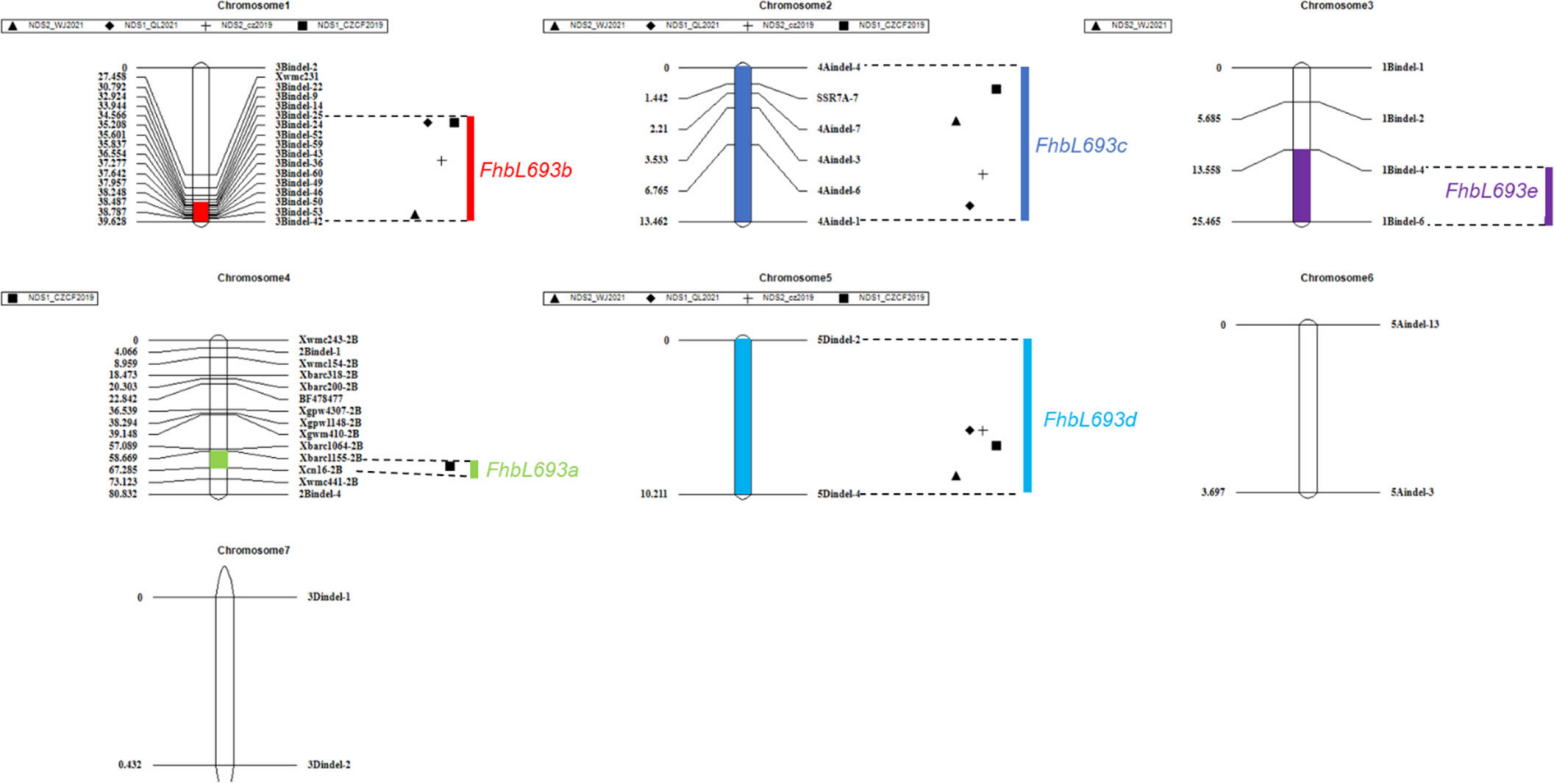
QTLs for *Fusarium* head blight on seven linkage groups in four places/year. Chromosomes 1, 2, 3, …, 7 represent chromosomes 3B, 4A, 1B, 2B, 5D, 5A and 3D, respectively. ▲, ◆, +, ■ respectively represent the FHB resistance QTLs detected in 2021WJ, 2021QL, 2019CZ^a^, 2019CZ^b^.

In this study, five QTLs were revealed. First, The QTL F*hbL693b* on chromosome 3B explained more of the phenotypic variation (**Table 6**) and was detected, so the QTL *FhbL693b* was precisely mapped using the linked markers (**Table 5**). The QTL *FhbL693b* was detected in all 4 environments. In 2019CZ^b^ and 2021QL, the *FhbL693b* QTL was narrowed to 0.64 cM and flanked by *3Bindel-25* and *3Bindel-24*; in 2021WJ, the *FhbL693b* QTL was mapped to 0.84 cM and flanked by *3Bindel-53* and *3Bindel-36*; in 2019CZ, the QTL *FhbL693b* was mapped to 0.72 cM and flanked by *3Bindel-43* and *3Bindel-36*. Combined with the mapping results of *FhbL693b*, the *FhbL693b* QTL was narrowed to 5.1 cM, approximately 49 Mb flanked by marker *3Bindel-25* (328,489,242 bp), *3Bindel-24* (331,950,894 bp)*, 3Bindel-53* (329,472,285 bp), *3Bindel-42* (302,784,826 bp)*, 3Bindel-43* (305,658,046 bp), or *3Bindel-36* (282,358,935 bp) in wheat reference genome v1.0, which could explain approximately 2.32~8.65% (4.99%, 7.26%, 8.65%, 2.32%) of the PVE and -0.15% ~ -0.77% (-0.1528%, -0.1988%, -0.7739%, -0.2221%) additive effect in 2021WJ, 2021QL, 2019CZ^a^ and 2019CZ^b^, respectively (**Table 6**; **Figure 5**).

**Table 6:** QTL information and phenotypic variation explanation (PVE).

QTL	Trait name	Chr.	Position/cM	Left marker	Right marker	LOD	PVE	Add
*FhbL693a*	NDS1_CZCF2019	2B	64	*Xbarc1155-2B*	*Xcn16-2B*	2.1122	1.2927	0.1649
*FhbL693b*	NDS2_WJ2021	3B	39	*3Bindel-53*	*3Bindel-42*	5.4716	4.9971	-0.1528
	NDS1_QL2021	3B	35	*3Bindel-25*	*3Bindel-24*	8.1246	7.2696	-0.1988
	NDS2_cz2019	3B	37	*3Bindel-43*	*3Bindel-36*	9.4571	8.6469	-0.7739
	NDS1_CZCF2019	3B	35	*3Bindel-25*	*3Bindel-24*	4.4402	2.3222	-0.2221
*FhbL693c*	NDS2_WJ2021	4A	2	*SSR7A-7*	*4Aindel-7*	3.7532	3.3116	0.1407
	NDS1_QL2021	4A	10	*4Aindel-4*	*4Aindel-1*	5.1115	5.266	0.1825
	NDS2_cz2019	4A	5	*4Aindel-3*	*4Aindel-6*	3.5861	3.3444	0.5308
	NDS1_CZCF2019	4A	1	*4Aindel-4*	*SSR7A-7*	3.6184	1.8959	0.2244
*FhbL693d*	NDS2_WJ2021	5D	9	*5Dindel-2*	*5Dindel-4*	10.4138	10.4757	0.2229
	NDS1_QL2021	5D	6	*5Dindel-2*	*5Dindel-4*	8.9024	9.3907	0.2257
	NDS2_cz2019	5D	6	*5Dindel-2*	*5Dindel-4*	9.699	10.4152	0.8533
	NDS1_CZCF2019	5D	7	*5Dindel-2*	*5Dindel-4*	4.3307	2.5422	0.2326
*FhbL693e*	NDS2_WJ2021	1B	23	*1Bindel-4*	*1Bindel-6*	2.1526	2.2942	-0.1036

NDS_CZ2019, the number of diseased spikelets in Chongzhou in 2019; NDS_CZCF2019, the number of diseased spikelets in Chongzhou replicate in 2019; NDS_WJ2021, the number of diseased spikelets in Wenjiang in 2021; NDS_QL2021, the number of diseased spikelets in Qionglai in 2021; Chr., chromosome; LOD, likelihood of odds; PVE, phenotypic variation explained by the marker; Add, estimated additive effect of the marker. The positions of QTLs refer to the genetic map in **Figure 5**.

Second, the *FhbL693c* QTL on chromosome 4A was detected in all 4 environments, and the location was narrowed to 13.462 cM, approximately 208 Mb, flanked by *SSR7A-7*, *4Aindel-7* (236,872,635 bp), *4Aindel-3* (58,532,210 bp), *4Aindel-6* (180,729,886), *4Aindel-4* (65,633,552 bp) and *4Aindel-1* (27,670,704 bp), which could explain approximately 1.89~5.26% (3.31%, 5.26%, 3.34%, 1.89%) of the PVE and 0.14~0.53% (0.1407%, 0.1825%, 0.5308%, 0.2244%) of the additive effect in 2021WJ, 2021QL, 2019CZ^a^ and 2019CZ^b^, respectively (**Table 6**; **Figure 5**).

Third, a novel QTL, *FhbL693d*, on chromosome 5D was detected in all 4 environments, and the location was narrowed to 10.2 cM, approximately 32 Mb, flanked by *5Dindel-2* (47,080,989 bp) and *5Dindel-4* (79,220,144 bp), which could explain approximately 2.54~10.48% (10.48%, 9.39%, 10.41%, 2.54%) of the PVE and 0.22~0.85% (0.2229%, 0.2257%, 0.8533%, 0.2326%) of the additive effect in 2021WJ, 2021QL, 2019CZ^a^ and 2019CZ^b^, respectively (**Table 6**; **Figure 5**).

Fourth, a minor effect QTL, *FhbL693a*, on chromosome 2BL was detected in one environment; this QTL was narrowed to 8.6 cM, flanked by *Xcn16-2B* and *Xwmc441-2B*, which could explain approximately 1.2% of the PVE and 0.16% of the additive effect in 2019CZ^b^ (**Table 6**; **Figure 5**).

Fifth, a minor effect QTL, *FhbL693e*, on chromosome 1B was detected in one environment; this QTL was narrowed to 11.9 cM, approximately 158 Mb, and flanked by *1Bindel-4* (374,519,369 bp) and *1Bindel-6* (532,662,331 bp), which could explain approximately 2.29% of the PVE and -0.10% of the additive effect in 2021WJ (**Table 6**; **Figure 5**).

### 3.5 Linked marker selection and the effect of QTLs on FHB resistance

In this study, five QTLs were found to be associated with FHB spread resistance. Among them, three QTLs on chromosomes 3B, 4A and 5D were detected in all the years/locations, while two minor QTLs on chromosomes 1B and 2B were detected in only one year/location. This finding illustrated that the QTLs on chromosomes 3B, 4A and 5D were stable and reliable. The linked markers of QTLs on chromosomes 3B, 4A and 5D were selected according to their close genetic proximity to the QTL locations. Three linked markers, *3Bindel-24*, *4Aindel-7* and *5Dindel-4*, were selected from QTLs *FhbL693b*, *FhbL693c* and *FhbL693d*, respectively.

**Figure 6:**
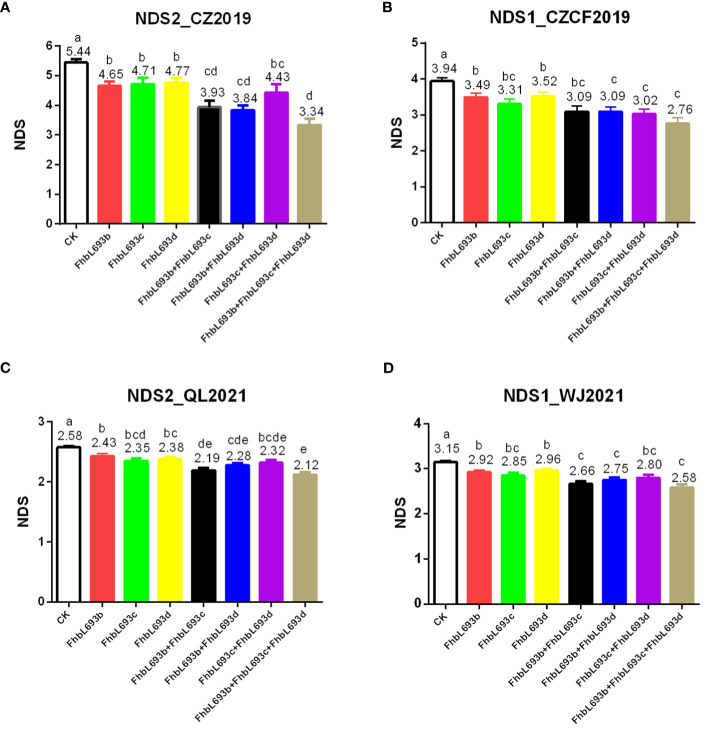
**(A–D)** respectively represent the effect of three QTL (FhbL693b, FhbL693c and FhbL693d) on NDS in 2019CZ^a^, 2019CZ^b^, 2021QL and 2021WJ. NDS2_CZ2019, the number of diseased spikelets in 2019CZ^a^; NDS1_CZCF2019, the number of diseased spikelets in 2019CZ^b^; NDS2_QL2021, the number of diseased spikelets in Qionglai in 2021; NDS1_WJ2021, the number of diseased spikelets in Wenjiang in 2021.

To determine the effect of QTLs on FHB spread resistance, we calculated the mean under three MAS conditions. The results showed that each QTL could significantly (P<0.05) decrease the NDS after MAS in 4 year/location field experiments (**Figure 6**), illustrating that the effects of the three QTLs were relatively greater and more stable. In total, these QTLs decreased the NDS by 2.1 at most after MAS decreased NDS by 39% in the FHB heavy conditions.

### 3.6 Screening of the FHB-resistant line

To increase the application speed of the three QTLs, three linked markers, *3Bindel-24*, *4Aindel-7* and *5Dindel-4*, were used to select lines with higher FHB resistance. After screening the three markers, the NDS in the F_10_ population significantly (P<0.05) decreased to 2.12-3.34, and 26 wheat lines were selected as described below. Among them, 20 wheat lines, especially H140-2, exhibited stronger FHB resistance (**Table 7**), immune to powdery mildew and moderately resistant to strip rust.

**Table 7:** FHB-resistant lines selected by the linked markers *3Bindel-24*, *4Aindel-7* and *5Dindel-4*.

Number	Genotype	NDS2_cz2019	NDS1_CZCF2019	NDS2_WJ2021	NDS1_QL2021
1	140-2	2	2	2	2
2	78-2	5.9	2.7	3	2.7
3	190-2	2.3	2.4	2	2.3
4	309-2	3.8	2.4	2.1	2
5	320-2	3.8	2.5	2.3	2.2
6	2-2	3.2	NA	2	3.3
7	3-2	2.6	3.5	2	2.5
8	6-2	NA	2.4	NA	NA
9	7-2	3.1	2.2	2.1	2.7
10	8-2	2.8	2.3	2.2	2.7
11	10-2	4	2.2	2.1	3.1
12	16-2	3.4	2.6	2.4	2.3
13	39-2	3.1	2.6	2	2.5
14	57-2	2.8	NA	2	3
15	79-2	2.7	2.5	2.2	2.7
16	98-2	6.4	2.1	2	2.4
17	105-2	5.7	NA	2.5	2.5
18	120-2	4.2	3.6	2.2	2.7
19	141-2	3.4	2.8	2	2.3
20	142-2	2.9	3.3	2	2.9
21	143-2	3.6	2.4	2	2
22	183-2	2.2	NA	2	2.2
23	189-2	2.1	2.3	2.1	2.3
24	249-2	2.6	5.6	2	3.4
25	288-2	2.5	NA	2	2.7
26	305-2	2.5	3	2	2.5

NDS2_CZ2019, the number of diseased spikelets in Chongzhou in 2019; NDS1_CZCF2019, the number of diseased spikelets in Chongzhou replicate in 2019; NDS2_WJ2021, the number of diseased spikelets in Wenjiang in 2021; NDS1_QL2021, the number of diseased spikelets in Qionglai in 2021.

## 4 Discussion

### 4.1 Three novel FHB resistance QTLs discovered in PI672538

FHB resistance is controlled by multiple genes and is easily affected by the environment. In this study, five QTLs were mapped in the RIL populations derived from the L661/PI672538 cross; these QTLs were mapped to chromosomes 1B, 2B, 3B, 4A, and 5D.

The QTL *FhbL693b* was mapped to chromosome 3B at 282~331 Mb (IWGSC Ref Seq v1.0) and was narrowed to 5.1 cM, which could explain approximately 2.3~8.6% of the PVE. FHB resistance QTLs on chromosome 3B were reported and derived from Sumai 3, Wangshuibai, Ernie, Truman, Huangfangzhu, and Baishanyuehuang (Liu et al., 2007; Zhang et al., 2012; Islam et al., 2016; Su et al., 2019). Sumai 3 and Wangshuibai carried the *Fhb1* QTL located on chromosome 3B (8.5 Mb; Gene ID: MK450312.1, IWGSC Ref Seq v1.0), and could explain approximately 30% of the PVE (Li et al., 2019; Su et al., 2019). The PVE and physical position of *Fhb1* are different from those of *FhbL693b* on chromosome 3B. This finding illustrated that *FhbL693b* is different from *Fhb1*. A small QTL was reported on chromosome 3BL, flanked by *Xcfa2134b* ~ *Xgwm3134b*, which could explain 6~9% of the PVE (Paillard et al., 2004); this QTL is different from that of the QTL *FhbL693b* because they have different physical positions. A QTL linked with *Xwmc615* derived from Truman on chromosome 3BSc could explain 7.3% of the PVE (Islam et al., 2016) and is similar to *FhbL693b* because they have similar physical positions and PVEs, although they have different pedigrees. Another QTL derived from Baishanyuehuang, named *Qfhb.hwwg-3BSc*, which is flanked by *Xwmc307*, *Xwmc366* and *Xgwm566*, could explain 8.5% of the PVE (Zhang et al., 2012); this QTL is also similar to *FhbL693b* because they have similar physical positions and PVE. In the present study, *FhbL693b* was similar to the QTLs derived from Baishanyuehuang and Truman (Zhang et al., 2012; Islam et al., 2016). This illustrated that the *FhbL693b* QTL was present. Furthermore, the *FhbL693b* QTL was narrowed to 5.1 cM (282~331 Mb), a location that is more precise than that of the QTLs derived from Baishanyuehuang and Truman (Zhang et al., 2012; Islam et al., 2016). In addition, a previous study reported that *FhbL693b* was narrowed to 18.01 cM and flanked by *Xwmc54-3B* and *Xgwm566-3B* (Li et al., 2017). In this study, *FhbL693b* was narrowed to 5.1 cM, which was further narrowed to a more precise location than was found in a previous study (Li et al., 2017).

The FHB spread resistance QTL on chromosome 5D was previously reported to be derived from Wangshuibai and flanked by *Xbarc322* and *Xgwm97*; this QTL could explain 5.5% of the PVE (Yu et al., 2008). The physical position of the marker *Xbarc322* (sequence ID: BV211665) was 497,832,712 bp ~ 497,833,051 bp. In this study, a QTL on chromosome 5D, *FhbL693d*, was detected at 4 locations/year and narrowed to 10.2 cM, about 32 Mb, which could explain approximately 10.5% of the PVE; this QTL was flanked by *5Dindel-2* (47,080,989 bp) and *5Dindel-4* (79,220,144 bp). *FhbL693d* was different from the QTL derived from Wangshuibai (Yu et al., 2008) because of their different physical positions. Therefore, the *FhbL693d* QTL is a novel QTL.

An FHB spread resistance QTL on chromosome 4A was previously reported that was derived from the cross of Arina/Forno and flanked by *Xcdo545 ~ Xgwm160* on chromosome 4AL (Paillard et al., 2004). In this study, a QTL, *FhbL693c*, located on chromosome 4A, flanked by *4Aindel-1* (27,670,704 bp) and *4Aindel-7* (236,872,635 bp) on chromosome 4AS explained 1.9%~5.2% of the PVE. This QTL is different from the QTL derived from Arina/Forno because the two QTLs are located in different chromosomal regions. Therefore, the QTL *FhbL693c* is a novel QTL.

A previous study mapped the FHB resistance QTL *FhbL693a*, which was flanked by the markers *Xcn16-2B* and *Xwmc441-2B*, in the F_2_ population derived from the cross of L661/PI672538 (Li et al., 2017). In this study, the QTL *FhbL693a* was also detected in one year/location in the F_10_ RIL population, which was derived from the same cross. This finding illustrated that the QTL *FhbL693a* truly existed, although its effect was smaller in this study than in previous studies (Li et al., 2017). The decrease in the PVE of *FhbL693a* in the RIL population in this study may have been caused by the change in the mapping population.

Previous researches reported two FHB spread resistance QTLs mapped to chromosome 1B. One QTL on chromosome 1BS was derived from Alondra’s’ and flanked by *XEtcg.Magc-7* – *XEaccg.Mctc-7*; this QTL could explain 15.6% of the PVE (Zhang et al., 2004; Buerstmayr et al., 2010). In this study, a minor effect QTL, *FhbL693e*, on chromosome 1B was detected in one location; this QTL was narrowed to 11.9 cM, approximately 158 Mb, and flanked by *1Bindel-4* (374,519,369 bp) and *1Bindel-6* (532,662,331 bp), which could explain approximately 2.29% of the PVE and -0.10% of the additive effect in 2021WJ. The QTL on 1BS derived from Alondra’s’ is different from the QTL *FhbL693e* in this study because they have different physical locations and PVEs. Another QTL on chromosome 1BS, derived from F201R and associated with *Xbarc8* (46,893,462 bp), could explain 16% of the PVE (Shen et al., 2003); this QTL is different from the *FhbL693e* QTL in this study because they have different physical positions and PVEs. This finding illustrated that *FhbL693e* is a novel QTL.

### 4.2 The RIL population was more effective at QTL mapping than the F_2_ population

In this study, the correlation coefficient of NDS in 2014-2017 in the F_2:7_ population was very weak (**Supplementary Table 1**). However, the correlation coefficient of NDS in F_10_ during 2019-2021 was obviously greater than that in the F_2:7_ population (**Table 2**; **Supplementary Table 1**). This finding illustrated that FHB resistance in the RIL population was more stable than that in the F_2:7_ population. Therefore, the RIL population is more suitable for QTL mapping than the F_2:7_ population and the RIL F_10_ population was used for QTL mapping in this study. A previous study using an F_2_ population and an F_2:3_ family showed that the FHB resistance of PI672538 is controlled by two major QTLs (*QfhbL693a* and *QfhbL693b*) (Li et al., 2017). However, in the present study, QTL mapping via the RIL population revealed that FHB resistance in PI672538 was controlled by five QTLs, including *QfhbL693a* and *QfhbL693b*. This finding illustrated that the RIL population could harbor more QTLs than the F_2_ population and F_2:3_ family. A previous study also showed that more QTLs were detected in the RIL population than in the F_2_ population (Tang et al., 2000), which further supported the finding that the RIL population was more effective at QTL mapping than the F_2_ population and F_2:3_ family.

### 4.3 Multiple QTLs make PI672538 resistant to FHB

Although no major effect QTL was detected for PI672538, this strain still exhibited stronger FHB resistance in this study. Five QTLs were detected in PI672538 that confer resistance to FHB. Among them, three QTLs significantly decrease NDS caused by FHB (**Figure 6**), and they could decrease NDS by 39% in the F_10_ population after MAS. These findings illustrated that these three QTLs are important for the construction of FHB-resistant PI672538 strains.

### 4.4 Potential utilization of the FHB resistance QTLs of PI672538 in wheat breeding practice

Sumai 3, Wangshuibai, and their derivatives are well-known sources of FHB resistance, and the major resistance QTL *Fhb1* is located on chromosome arm 3BS (Li et al., 2019; Su et al., 2019). However, it is difficult to use these sources in wheat breeding because they have many undesirable agronomic traits. In our studies, PI672538 was highly resistant to stripe rust, powdery mildew and FHB (Liu et al., 2015; Li et al., 2017; Huang and Luo, 2021). Therefore, PI672538 could play a key role in improving wheat resistance. Furthermore, the high FHB resistance of PI672538 is controlled by five QTLs, which makes PI672538 an important FHB resistance source. PI672538 and its derivatives have been widely used in wheat breeding in Henan, Shandong, Beijing, Shanxi and Sichuan Provinces (Li et al., 2017). We believe that many cultivars will be developed and identified with QTLs derived from PI672538 and its derivatives in the future.

## 5 Conclusions

Total five FHB resistance QTLs were detected in PI672538 by bulked segregant analysis sequencing of recombinant inbred line population, including previously reported two QTL (*FhbL693a* and *FhbL693b*). Two major QTLs (*FhbL693c* and *FhbL693d*) and a minor QTL (*FhbL693e*) were first reported in this study. Three QTLs *FhbL693b*, *FhbL693c* and *FhbL693d* could significantly decrease the number of *Fusarium*-damaged spikelets. The selection of *FhbL693d*, *FhbL693c* and *FhbL693b* could at most decrease the number of damaged spikelets by 2.1 (39%), and a new line H140-2 resistant to FHB was developed by marker-assisted selection (MAS). This results in our study would help for wheat FHB resistance improvement.

## Data availability statement

The data presented in the study are deposited in the National genomics Data Center (NGDC) repository, accession number CRA017199.

## Author contributions

QH: Data curation, Formal analysis, Funding acquisition, Investigation, Methodology, Project administration, Software, Supervision, Validation, Visualization, Writing – original draft, Writing – review & editing. XL: Funding acquisition, Investigation, Writing – original draft. QL: Funding acquisition, Investigation, Writing – original draft. SZ: Investigation, Writing – original draft. XYL: Investigation, Writing – original draft. JY: Investigation, Writing – original draft. FT: Investigation, Writing – original draft. TR: Conceptualization, Resources, Writing – review & editing. ZL: Conceptualization, Writing – review & editing. YS: Funding acquisition, Project administration, Writing – review & editing.

## Supplementary material

Supplementary Table 1 the correlation analysis of the NDS of F_2:7_ RILs population during 2015-2017 wheat growing season. NDS201528WJ, the number of diseased spikelets in Wenjiang in 2015; NDS201619NJ, the number of diseased spikelets in Neijiang in 2016; NDS201628WJ, the number of diseased spikelets in Wenjiang in 2016; NDS2017FL, the number of diseased spikelets in Fuling in 2017; NDS2017WJ, the number of diseased spikelets in Wenjiang in 2017; NDS2017NJ, the number of diseased spikelets in Neijiang in 2017. ^**,^ the correlation index is significant at P<0.01 level.

Supplementary Table 2 FHB resistance evaluation of R and S pool in 2017 and 2018 years. NDS-FL, the number of diseased spikelets in Fuling; PDS-FL, the percent of diseased spikelets in Fuling; NDS-WJ, the number of diseased spikelets in Wenjiang; PDS-WJ, the percent of diseased spikelets in Wenjiang; NDS-NJ, the number of diseased spikelets in Neijiang; PDS-NJ, the percent of diseased spikelets in Neijiang.

Supplementary Table 3 The primer sequence of developed interval molecular markers. Chr., chromosome; Size, PCR amplification fragment size.
