# Supplementary material for: Three novel QTLs for FHB resistance identified and mapped in spring wheat PI672538 by bulked segregant analysis of the recombinant inbred line
Source: Front Plant Sci. 2024 Jul 29;15:1409095. doi: 10.3389/fpls.2024.1409095 (PMC11317384; doi:10.3389/fpls.2024.1409095)
Supplement: Supplementary Table 1 — the correlation analysis of the NDS of F2:7 RILs population during 2015-2017 wheat growing season. NDS201528WJ, the number of diseased spikelets in Wenjiang in 2015; NDS201619NJ, the number of diseased spikelets in Neijiang in 2016; NDS201628WJ, the number of diseased spikelets in Wenjiang in 2016; NDS2017FL, the number of diseased spikelets in Fuling in 2017; NDS2017WJ, the number of diseased spikelets in Wenjiang in 2017; NDS2017NJ, the number of diseased spikelets in Neijiang in 2017. **, the correlation index is significant at P<0.01 level. [file Table_1.docx]

Supplementary Table 1. the correlation analysis of the NDS of F_2:7_ RILs population during 2015-2017 wheat growing season.

|  | NDS201528WJ | NDS201619NJ | NDS201628WJ | NDS2017FL | NDS2017WJ | NDS2017NJ |
| --- | --- | --- | --- | --- | --- | --- |
| NDS201528WJ | 1 |  |  |  |  |  |
| NDS201619NJ | 0.210^**^ | 1 |  |  |  |  |
| NDS201628WJ | 0.306^**^ | 0.191^**^ | 1 |  |  |  |
| NDS2017FL | 0.055 | -0.033 | 0.072 | 1 |  |  |
| NDS2017WJ | 0.172^**^ | 0.032 | 0.185^**^ | 0.331^**^ | 1 |  |
| NDS2017NJ | 0.055 | -0.061 | 0.053 | -0.082 | 0.206^**^ | 1 |

NDS201528WJ, the number of diseased spikelets in Wenjiang in 2015; NDS201619NJ, the number of diseased spikelets in Neijiang in 2016; NDS201628WJ, the number of diseased spikelets in Wenjiang in 2016; NDS2017FL, the number of diseased spikelets in Fuling in 2017; NDS2017WJ, the number of diseased spikelets in Wenjiang in 2017; NDS2017NJ, the number of diseased spikelets in Neijiang in 2017. ^**,^ the correlation index is significant at P<0.01 level.
