# Supplementary material for: Three novel QTLs for FHB resistance identified and mapped in spring wheat PI672538 by bulked segregant analysis of the recombinant inbred line
Source: Front Plant Sci. 2024 Jul 29;15:1409095. doi: 10.3389/fpls.2024.1409095 (PMC11317384; doi:10.3389/fpls.2024.1409095)
Supplement: Supplementary Table 2 — FHB resistance evaluation of R and S pool in 2017 and 2018 years. NDS-FL, the number of diseased spikelets in Fuling; PDS-FL, the percent of diseased spikelets in Fuling; NDS-WJ, the number of diseased spikelets in Wenjiang; PDS-WJ, the percent of diseased spikelets in Wenjiang; NDS-NJ, the number of diseased spikelets in Neijiang; PDS-NJ, the percent of diseased spikelets in Neijiang. [file Table_2.docx]

Supplementary Table 2. FHB resistance evaluation of R and S pool in 2017 and 2018 years.

| Pool | Number (F_10_) | 2017 | | | | | | 2018  NDS |
| --- | --- | --- | --- | --- | --- | --- | --- | --- |
|  |  | NDS-FL | PDS-FL | NDS-WJ | PDS-WJ | NDS-NJ | PDS-NJ |  |
| R pool | 20 | 5.75 | 0.36 | 2.00 | 0.11 | 3.78 | 0.29 | 2.00 |
|  | 50 | 3.00 | 0.16 | 2.50 | 0.12 | 2.90 | 0.15 | 2.00 |
|  | 58 | 7.63 | 0.45 | 2.67 | 0.15 | 2.36 | 0.14 | 2.00 |
|  | 61 | 2.13 | 0.13 | 3.00 | 0.14 | 2.00 | 0.10 | 2.00 |
|  | 64 | 2.25 | 0.10 | 3.00 | 0.14 | 2.20 | 0.12 | 2.25 |
|  | 78 | 2.71 | 0.14 | 2.56 | 0.14 | 2.13 | 0.13 | 2.00 |
|  | 86 | 2.25 | 0.12 | 2.18 | 0.10 | 2.13 | 0.10 | 2.00 |
|  | 95 | 2.43 | 0.16 | 2.60 | 0.14 | 2.00 | 0.10 | 2.00 |
|  | 98 | 2.80 | 0.15 | 2.50 | 0.14 | 2.11 | 0.14 | 2.20 |
|  | 107 | 3.80 | 0.19 | 2.22 | 0.12 | 2.10 | 0.16 | 2.00 |
|  | 117 | 2.29 | 0.13 | 2.56 | 0.13 | 2.83 | 0.17 | 2.20 |
|  | 120 | 2.40 | 0.14 | 2.10 | 0.11 | 2.63 | 0.16 | 2.00 |
|  | 140 | 2.50 | 0.15 | 2.50 | 0.13 | 2.43 | 0.17 | 2.50 |
|  | 150 | 2.88 | 0.14 | 2.20 | 0.10 | 2.00 | 0.10 | 2.00 |
|  | 179 | 2.63 | 0.13 | 2.80 | 0.13 | 2.30 | 0.14 | 2.00 |
|  | 205 | 2.40 | 0.13 | 2.40 | 0.13 | 2.20 | 0.12 | 2.00 |
|  | 208 | 2.40 | 0.13 | 2.44 | 0.13 | 2.00 | 0.11 | 2.00 |
|  | 225 | 2.50 | 0.13 | 2.10 | 0.10 | 2.10 | 0.16 | 2.38 |
|  | 227 | 2.78 | 0.15 | 2.20 | 0.11 | 2.22 | 0.14 | 2.56 |
|  | 281 | 4.00 | 0.21 | 2.80 | 0.15 | 2.10 | 0.13 | 2.00 |
|  | 310 | 2.57 | 0.14 | 2.29 | 0.12 | 2.50 | 0.17 | 2.00 |
|  | 313 | 2.22 | 0.11 | 2.11 | 0.11 | 2.00 | 0.13 | 2.00 |
|  | 326 | 2.57 | 0.14 | 2.44 | 0.13 | 2.10 | 0.14 | 2.00 |
|  | 336 | 2.78 | 0.15 | 2.67 | 0.13 | 2.70 | 0.18 | 2.00 |
|  | 341 | 2.70 | 0.16 | 2.50 | 0.13 | 2.67 | 0.19 | 2.00 |
|  | 355 | 2.20 | 0.13 | 2.43 | 0.14 | 2.78 | 0.14 | 2.00 |
| S pool | 5 | 4.20 | 0.23 | 4.80 | 0.27 | 3.40 | 0.26 | 6.50 |
|  | 116 | 4.33 | 0.24 | 2.71 | 0.14 | 2.67 | 0.18 | 6.50 |
|  | 209 | 2.64 | 0.15 | 6.33 | 0.35 | 3.10 | 0.19 | 6.71 |
|  | 237 | 5.45 | 0.32 | 5.67 | 0.30 | 3.33 | 0.20 | 7.00 |
|  | 242 | 5.38 | 0.34 | 4.70 | 0.24 | 2.20 | 0.12 | 6.25 |
|  | 243 | 4.11 | 0.21 | 6.44 | 0.32 | 2.00 | 0.11 | 11.38 |
|  | 252 | 3.88 | 0.22 | 4.80 | 0.30 | 4.33 | 0.27 | 6.57 |
|  | 254 | 5.50 | 0.31 | 3.89 | 0.22 | 2.67 | 0.17 | 7.43 |
|  | 255 | 3.30 | 0.17 | 5.44 | 0.27 | 4.60 | 0.24 | 8.29 |
|  | 256 | 2.56 | 0.13 | 2.89 | 0.14 | 4.60 | 0.21 | 10.5 |
|  | 263 | 6.91 | 0.36 | 6.25 | 0.39 | 2.70 | 0.13 | 7.50 |
|  | 271 | 3.11 | 0.16 | 3.63 | 0.18 | 2.20 | 0.10 | 9.43 |
|  | 272 | 3.00 | 0.17 | 2.56 | 0.14 | 2.57 | 0.18 | 9.00 |
|  | 274 | 3.70 | 0.21 | 4.70 | 0.24 | 2.10 | 0.12 | 11.57 |
|  | 276 | 2.78 | 0.14 | 4.56 | 0.24 | 3.11 | 0.22 | 8.00 |
|  | 285 | 8.60 | 0.48 | 4.82 | 0.30 | 2.20 | 0.16 | 6.29 |
|  | 296 | 5.00 | 0.26 | 4.22 | 0.26 | 3.20 | 0.19 | 6.80 |
|  | 240 | 3.00 | 0.15 | 3.00 | 0.14 | 2.20 | 0.12 | 11.67 |
|  | 259 | 2.80 | 0.16 | 2.56 | 0.13 | 3.40 | 0.16 | 8.71 |
|  | 273 | 3.11 | 0.16 | 3.40 | 0.17 | 3.00 | 0.16 | 9.63 |

NDS-FL, the number of diseased spikelets in Fuling; PDS-FL, the percent of diseased spikelets in Fuling; NDS-WJ, the number of diseased spikelets in Wenjiang; PDS-WJ, the percent of diseased spikelets in Wenjiang; NDS-NJ, the number of diseased spikelets in Neijiang; PDS-NJ, the percent of diseased spikelets in Neijiang.
