# Supplementary material for: Three novel QTLs for FHB resistance identified and mapped in spring wheat PI672538 by bulked segregant analysis of the recombinant inbred line
Source: Front Plant Sci. 2024 Jul 29;15:1409095. doi: 10.3389/fpls.2024.1409095 (PMC11317384; doi:10.3389/fpls.2024.1409095)
Supplement: Supplementary Table 3 — The primer sequence of developed interval molecular markers. Chr., chromosome; Size, PCR amplification fragment size. [file Table_3.docx]

Supplementary Table 3. The primer sequence of developed interval molecular markers.

| **Marker name** | **Chr.** | **Start position** | **End position** | **Size** | **F** | **R** | **Type** |
| --- | --- | --- | --- | --- | --- | --- | --- |
| *1Aindel-1* | 1A | 23079404 | 23080204 | 246 | TTCTGGGCCACAATTCCAGG | GTTCCAACAAAGTGGGCTGG | InDel |
| *ms1A-8* | 1A | 415171658 | 415172669 | 212 | ATGCCGCCAATTCCTCTTGA | TCTCTCTGTATGCGGGGCTA | SSR |
| *ms1A-9* | 1A | 415261497 | 415262547 | 327 | CCCTTCCTCTTCGGGGAAAA | AGATAAGGGAGAAGATAAGGGAC | SSR |
| *ms1A-11* | 1A | 415298254 | 415299271 | 278 | CCAGGAAGGATCGCAGTTCT | CAACCATGGCCAGTTTCAGGA | SSR |
| *ms1A-10* | 1A | 415305657 | 415306674 | 557 | AGGCCCCGATTAGGAGTACA | GAGGATCTGGCCGACAAAGT | SSR |
| *ms1A-1* | 1A | 510452658 | 510453669 | 523 | TGGAGTCAGGAGTGTGTTCG | TGCGTTCATGGATGCACTTC | SSR |
| *ms1A-2* | 1A | 510621591 | 510622679 | 595 | TAACGGCCGGAATCCTCGTC | CACTACCGCCCCCATGAAC | SSR |
| *ms1A-3* | 1A | 510761224 | 510762233 | 321 | GCCACTGCTACCAGATATAG | TAGCTGCTCTCAGGTTACCA | SSR |
| *ms1A-4* | 1A | 510857350 | 510858359 | 294 | CGGCTTCAAAACCTTTTCGTGT | AACGGACCTCCAGGTTGTGA | SSR |
| *ms1A-5* | 1A | 510963143 | 510964160 | 575 | CTGATTCCGATAAACGAGCGAA | ATAAGCCGGGGCAAGCCA | SSR |
| *ms1A-6* | 1A | 511006809 | 511007822 | 421 | CCTTCGGAGGCACGTTTCAT | TTTCATCATGTCCGTTGCGTG | SSR |
| *ms1A-7* | 1A | 511102270 | 511103287 | 347 | AGTTCCATAGCACGCACGAG | GCTGTAGTTGGTTGGGACCTC | SSR |
| *1Bindel-1* | 1B | 121730066 | 121730866 | 262 | AAGCACATTGCACCCTACGA | ATGGTCTGCCTCTACCCTGT | InDel |
| *1Bindel-2* | 1B | 142612223 | 142613023 | 329 | GCTGGTAGCTTTGCATTTGCT | ACCGGTTGGAATCCGAGGAG | InDel |
| *1Bindel-3* | 1B | 334285435 | 334286235 | 291 | GAGAGTTTCCCCTCTGGTCC | GGTTTCCTTGTGGGCAACTC | InDel |
| *1Bindel-4* | 1B | 374519369 | 374520169 | 217 | GGGGATTTAGTTGCACGGGA | GCCCTCAGTGGTGTCTTTGT | InDel |
| *1Bindel-5* | 1B | 505796101 | 505796901 | 329 | GGAGCCATCTCTACACCACC | AAGAATACATGCGCTCAGCA | InDel |
| *1Bindel-6* | 1B | 532662331 | 532663131 | 230 | ATGTTACTGTGCAACGCCGA | TCATTCGCGGCAGGAAGTT | InDel |
| *1Dindel-1* | 1D | 39651903 | 39652703 |  | TGGCAAACTGCCCTATCATCA | AGTTCCAAAACCAGTGGCTG | InDel |
| *1Dindel-2* | 1D | 158016084 | 158016884 | 233 | CCCCAAGCCCAATCCGAA | AAACGTTGGTGTGCCTTTGG | InDel |
| *1Dindel-3* | 1D | 254439567 | 254440367 | 347 | TCTAGGGCTTCGTGAGTATGA | CCGCGGGGTGTATGGC | InDel |
| *1Dindel-4* | 1D | 255062690 | 255063490 | 316 | ATGTGTGTGATACGCGGC | AGTTTACTTTATTGGTGTGTGGAAA | InDel |
| *2Aindel-1* | 2A | 407296997 | 407297797 | 319 | CGGTGATCGCTCTCACAGGG | GGTCGCAACAACCGGGTACA | InDel |
| *2Aindel-2* | 2A | 464707990 | 464708790 | 341 | GGTGAGTTAGCTGGGCTGTG | TCCCACCCTTTTTGTTTTGGG | InDel |
| *2Aindel-3* | 2A | 738410484 | 738411284 | 191 | GAGAAGTCATTGCCCCACCA | GGCACTACCAAGCACCTCTT | InDel |
| *ms2A-1* | 2A | 739268754 | 739269768 | 509 | GGGACTACGAACAACTTTCGGGATA | GCTGATGCCTCTACGTTTTCTTC | SSR |
| *ms2A-2* | 2A | 739312253 | 739313267 | 571 | TCGATAGATCCGGGGGCAG | AGAAGTAGAAAGTGCGCCAG | SSR |
| *2Bindel-1* | 2B | 44583441 | 44584241 | 335 | TGCGAAAGATTCTCCCGAAGG | CATGGAACCGACAACAGGC | InDel |
| *2Bindel-2* | 2B | 571881382 | 571882182 | 277 | ACTGCACGTGTGCCTTTTGT | ATTTGTTTCTCGTGGAGGTGC | InDel |
| *2Bindel-3* | 2B | 584320281 | 584321081 | 327 | CGGAGCCCAATGTGTGGTAG | GCATGACCTGTCATTGCCAC | InDel |
| *2Bindel-4* | 2B | 585883664 | 585884464 | 334 | GTTGTCAAGAGCTTTCCAACGA | AGCAAACATAGTATTGGTCACTTG | InDel |
| *2Bindel-5* | 2B | 740032358 | 740033158 | 223 | CGCACGCCCGAGATGTATTA | ATGGGGGCTGAAGCTGACA | InDel |
| *2Dindel-1* | 2D | 299993702 | 299994502 | 275 | TCTCACCCCCTCCGATTTTCA | ATGTTCAAGCCCGGAGTCAA | InDel |
| *3Aindel-1* | 3A | 522773456 | 522774256 | 292 | TCGTAAGTGGCCGTGTCTATC | AAGAGGTTGGGGGTAAGTGC | InDel |
| *3Aindel-2* | 3A | 532385470 | 532386270 | 282 | CCAGCCAAGAACAGACCCTC | TTTGGTTGCAGGGTTGCTTG | InDel |
| *3Aindel-3* | 3A | 611806096 | 611806896 | 343 | AGGGTATCTTACAGACCCGAC | TTGAGGGGCTCTTCAAGCTG | InDel |
| *3Aindel-4* | 3A | 719593122 | 719593922 | 266 | ACCTTGCTGCAAAAGGTGGA | AGCAGGATTGCGTACGTTGT | InDel |
| *3Bindel-2* | 3B | 29852204 | 29853004 | 168 | AGTACATGGTGGGTGACTGC | AGGTCAAACTCACTGTCGGT | InDel |
| *3Bindel-3* | 3B | 40825737 | 40826537 | 300 | TGATGAAACATGTGTCTACGGTGA | AGCGTCGCTATACGACCAAC | InDel |
| *3Bindel-4* | 3B | 68390138 | 68390938 | 255 | ACCCCCAAAACCCCTAAAGC | GGGGGTATGAACCATAAAGAACT | InDel |
| *3Bindel-5* | 3B | 95879250 | 95880050 | 252 | CCACTGGAATTTGCGTGAGC | GTGGCTCAACCTCTGTAGGC | InDel |
| *3Bindel-6* | 3B | 187685506 | 187686306 | 302 | TATACGGATCAAGGGGCCGA | TGTAGATCATCACCGCGAGC | InDel |
| *3Bindel-7* | 3B | 239111342 | 239112142 | 284 | CATGTAACAGGAGCTGGAAGC | TGTGGCTCAGGGAGAGGAT | InDel |
| *3Bindel-11* | 3B | 254368154 | 254368954 | 349 | AACTGATCCCATGTGACAAACTT | AGATCACCCAGTTGATCCAGC | InDel |
| *3Bindel-12* | 3B | 255924247 | 255925047 | 246 | GTCTAATCGGTGAACGGGGC | GCTCAGCTGCTCCAAAGACA | InDel |
| *3Bindel-13* | 3B | 256532581 | 256533381 | 235 | CGCGCCATAGAGAGGGC | CCAGCAGAGCTTTACGGGAG | InDel |
| *3Bindel-14* | 3B | 258355404 | 258356204 | 232 | ACTTACTGAGTATCCGCCCAC | CAGGATCAACTGACGATTGCG | InDel |
| *3Bindel-33* | 3B | 271146877 | 271147675 | 116 | CCATCGCTCCATCTCATGCT | CAGAGGACCAACCGCTTCTT | InDel |
| *3Bindel-34* | 3B | 274621461 | 274622259 | 400 | TTGTGGTCGCTACAACCTGG | CCGCGATACATCAAGATTGACA | InDel |
| *3Bindel-35* | 3B | 279645720 | 279646518 | 240 | AGTAATGCGGTGTACCCTGTC | ACACACATCGATGGCGACAA | InDel |
| *3Bindel-15* | 3B | 281642205 | 281643005 | 193 | GGAAACAATTGGGTTCCGATGC | CACTGGTACGTGTTGGTGCT | InDel |
| *3Bindel-36* | 3B | 282358935 | 282359733 | 211 | AGGAGGCCTCTTATCGTCCG | TGATAGAGAGGACACTCGCTCC | InDel |
| *3Bindel-37* | 3B | 284215018 | 284215816 | 226 | GCCTTGCCTCTGCCTACTGG | GGGGAGTTTTGGCACTGTTTTC | InDel |
| *3Bindel-16* | 3B | 284409868 | 284410668 | 327 | GAGAAGGGCCTGAAGACTTTGA | CTTCCGCAAGTGGTGTCCATA | InDel |
| *3Bindel-38* | 3B | 284489371 | 284490169 | 343 | GCTTCTCGTCTGTGCCTCTC | ACGCGGTATTTCGGTCTCTG | InDel |
| *3Bindel-17* | 3B | 287168332 | 287169132 | 382 | TGTCTTTATGACCCATGAACAGTTA | GGGTTTATCGCCACAACCTTCA | InDel |
| *3Bindel-39* | 3B | 292959358 | 292960156 | 354 | GATTCCGAGCGTGTCACCAT | TTAGTCAGTGGCCTAGGGGG | InDel |
| *3Bindel-18* | 3B | 293408819 | 293409619 | 376 | AAAGAAAGGTTCTGAAATCAACAGA | TCTAAACACGAGAGCGGTGG | InDel |
| *3Bindel-19* | 3B | 299032455 | 299033255 | 345 | GTCTTCGTCATCCGAGGAGC | GACCAACCCCGCGAGTTC | InDel |
| *3Bindel-20* | 3B | 299371977 | 299372777 | 279 | GATGAAACAAGTCCCCTGGC | ATCGGACTTTCGGACTGGC | InDel |
| *3Bindel-40* | 3B | 301053596 | 301054394 | 334 | GTTTTATCCTCCGTTGTGTCTTT | GGATATCTTCCAGCATTGGCG | InDel |
| *3Bindel-41* | 3B | 301546572 | 301547370 | 168 | TGTGATATGGAGAGAGAACGAAGA | GTCGCCCATAACTCTCTCACG | InDel |
| *3Bindel-42* | 3B | 302784826 | 302785624 | 180 | GTACCACGACCCACATCAGT | CCTTAGTACCGCAAAACCCGA | InDel |
| *3Bindel-43* | 3B | 305658046 | 305658844 | 302 | AGTCATAACGACCAAGGCCAG | AACTTCACACAAGCAACCACAC | InDel |
| *3Bindel-8* | 3B | 307028099 | 307028899 | 273 | CTATCGAGCAGAAGCCCTCG | CATGGCAGAAAGCCAGATGC | InDel |
| *3Bindel-21* | 3B | 307028099 | 307028899 | 231 | TATCGAGCAGAAGCCCTCGT | GCCTTGATTCCAAGAGCCGT | InDel |
| *3Bindel-44* | 3B | 307742565 | 307743363 | 362 | TTGGAGGCTGACTTGTAGGC | CCCATGCGAAGTGGTACGAG | InDel |
| *3Bindel-45* | 3B | 308250513 | 308251311 | 351 | GTGGGGTAACTACCACCGGG | GCAACGAATGCACTGACAGC | InDel |
| *3Bindel-46* | 3B | 311072382 | 311073180 | 119 | ACGATGAACGGAGTGTTGGT | CTGTCTGTGAGGCAGAGCTT | InDel |
| *3Bindel-47* | 3B | 317531765 | 317532563 | 390 | CATAGGTTTAACTAACAAGTACGCA | ATCCAGAGATTCGTCGACAC | InDel |
| *3Bindel-9* | 3B | 320622057 | 320622857 | 269 | CGGGACTACTGCAAGGGTAA | GTCCACGGCGAGCTTTTCTC | InDel |
| *3Bindel-22* | 3B | 320881284 | 320882084 | 337 | CGCATGACCTCCAGCATACTA | TGCTTGTCTATCATGCGTCC | InDel |
| *3Bindel-23* | 3B | 321594823 | 321595623 | - | GATAAAGGACAGCATTGGCATTGG | CTGGTATCGTTCCCAGGATGAG | InDel |
| *3Bindel-48* | 3B | 324182086 | 324182884 | 311 | GCATAAGCCCCCTGTCAAGT | AACATTAGCAAACCCACGCC | InDel |
| *3Bindel-49* | 3B | 325099648 | 325100446 | 214 | ACCGATAGGTGGGTCCTGTG | GCTTGCAAACTCAACCGCAA | InDel |
| *3Bindel-50* | 3B | 325684304 | 325685102 | 364 | CCACCGAAGCTCACTCAGTT | ATGTTCGGACAATGCCAGAT | InDel |
| *3Bindel-51* | 3B | 327305623 | 327306421 | 262 | ATTTTGGAACGGAGAGAGTGAA | CATCCGCATGTGGTTCATGG | InDel |
| *3Bindel-24* | 3B | 328489242 | 328490042 | 287 | ATGTGGAAGGGTGCGAGAAG | GATCATTGCACGCACCGAC | InDel |
| *3Bindel-52* | 3B | 328663127 | 328663925 | 309 | ACGGTGTTAGCGGAATAAGAGG | GGGTGAAAGAGAACAACGCAG | InDel |
| *3Bindel-53* | 3B | 329472285 | 329473083 | 366 | CAGAGCAGAACGTTCAAGATGC | CGACAGCGTCTCGTCTCAT | InDel |
| *3Bindel-25* | 3B | 331950894 | 331951694 | 326 | GCCCACACCATACGGTATCT | CGCTTGGACGGTTTTACGAA | InDel |
| *3Bindel-54* | 3B | 332694839 | 332695637 | 374 | GCCAAACAGGGAGGTTCAGG | TAACCCCTATCGCCCCAGTG | InDel |
| *3Bindel-26* | 3B | 339120023 | 339120823 | 174 | GGTGAGGGCCTCTGGTTTTA | GTGTTGGGACAGCTGGGTAA | InDel |
| *3Bindel-55* | 3B | 341114533 | 341115331 | 382 | TTGTGTTCCCCTCAAAACTACT | CATCGTGTGTCGAGTTGCTG | InDel |
| *3Bindel-27* | 3B | 341505074 | 341505874 | 299 | ATCCTCCTCCCCATCAGCAA | TGAGTGGACAGGTGCAACAT | InDel |
| *3Bindel-28* | 3B | 343826123 | 343826923 | 241 | GCAGGAACTTGAGAGCGGAG | ATGTGCAAGTGTCTGTCCACT | InDel |
| *3Bindel-29* | 3B | 347352487 | 347353287 | 309 | ACATTCAACAACGCCCAATCAA | TGATGTCCTCATCAGTACTCCCC | InDel |
| *3Bindel-56* | 3B | 353246555 | 353247353 | 185 | CTAGGGTTTCTGCCATCCCC | GACCGTGTTAGCTTGTGTGC | InDel |
| *3Bindel-57* | 3B | 358319642 | 358320440 | 238 | TGCGGCAAAACGCACTCTC | GCAGCAGAATACCTTGATGTAGC | InDel |
| *3Bindel-30* | 3B | 360206536 | 360207336 | 266 | TGGAAATTGTTAAGGGTCACTGGA | GGCAGGAGGTTGAAGACGGT | InDel |
| *3Bindel-58* | 3B | 364932160 | 364932958 | 390 | AGGACAACGGTGGGCG | TAGGATTTTTGGAAATGCCTAGC | InDel |
| *3Bindel-31* | 3B | 365124642 | 365125442 | 174 | ACAAAACAAAGACATCACTCCGA | ACATACTCGGGGTGTGTTACC | InDel |
| *3Bindel-32* | 3B | 365612945 | 365613745 | 249 | CGTTGCTAGCGGAGATGCTA | CTTTTACACGACGACTCGCA | InDel |
| *3Bindel-59* | 3B | 368096132 | 368096930 | 379 | CAATGGCGTTGCCTTAGCAT | AGGAACCCTAGCCCTACCAG | InDel |
| *3Bindel-60* | 3B | 368845559 | 368846357 | 390 | CAAGAGAAACATGCACCGCT | AATTTCAACAAGGGGGCTGGA | InDel |
| *3Dindel-1* | 3D | 38551060 | 38551860 | 198 | TCATAATCGTGGCGGGGTTATT | TTGGATTGCAGGTCACCACT | InDel |
| *3Dindel-2* | 3D | 38949397 | 38950197 | 278 | ATAGCTTGCGAAGTAGCTCCC | CGGTTTTGACACTGACACCG | InDel |
| *4Aindel-1* | 4A | 27670704 | 27671504 | 236 | GGTTACCACACCGGTTCCAA | GTTGGGTGGGGTAGATTCCG | InDel |
| *4Aindel-2* | 4A | 37591745 | 37592545 | 213 | ACACCTTCTCTGTCGAGCTT | CAAGGCCGAATCCCAGAAGA | InDel |
| *4Aindel-3* | 4A | 58532210 | 58533010 | 207 | CAGGCAGGTCAAAGAACGGA | GTGCCACCTAGCTTGGGTTA | InDel |
| *4Aindel-4* | 4A | 65633552 | 65634352 | 216 | GAATCTATGTGCACCGGGCT | GGCCAACCGTGATCCGAATA | InDel |
| *4Aindel-10* | 4A | 66648768 | 66649568 | 314 | TCCACTTCGAGTTTGACCTTC | TGAGATCTAGGCTGGGCTCT | InDel |
| *4Aindel-11* | 4A | 72013386 | 72014186 | 198 | AGGATGGGGCAAAAGGATGC | GGATCATGGATGCGAAGACCT | InDel |
| *4Aindel-12* | 4A | 72062875 | 72063675 | 265 | TAGAGCCGGACTTACACCGT | GGCATCTCCAATGGCAAACC | InDel |
| *4Aindel-5* | 4A | 80260308 | 80261108 | 246 | TGTCACTCTCGGTTCATTTCA | AGCACAAGTGTGGGAAAGGAT | InDel |
| *4Aindel-13* | 4A | 85745722 | 85746522 | 368 | AGACATGGCTGGACTGGTTT | ACATGTTTTGACAGCATTACGC | InDel |
| *4Aindel-14* | 4A | 91183313 | 91184113 | 270 | CCCGACCCAAAATCGGTCAA | AGGAGTGAGGCTACGATCCA | InDel |
| *4Aindel-15* | 4A | 98963934 | 98964734 | 148 | CAACCGATCGACTACAACCCA | CCCAACTGGGCTGGGTATC | InDel |
| *4Aindel-16* | 4A | 103512085 | 103512885 | 210 | GACGAACGGGTCCTCACAAA | CACCTCACCTTTCCAGTGGC | InDel |
| *4Aindel-17* | 4A | 104321743 | 104322543 | 270 | GTTCACTGGGAGCCACCAAA | ATTGTGCCCGTCAGTAGCAG | InDel |
| *4Aindel-18* | 4A | 107531713 | 107532513 | 350 | ATCAATGCCCGGACACCTTC | CCTGGGAAGTCTGTGGTATGA | InDel |
| *4Aindel-19* | 4A | 126436282 | 126437082 | 359 | AGTCCAATTCGGACCAACCC | AGGAAGGAAAAGTTTTTCGGTGT | InDel |
| *4Aindel-20* | 4A | 139713534 | 139714334 | 332 | CTGTTCCACTGGTGAGCGAT | TACTCCTGGTAGCGGTCGAA | InDel |
| *4Aindel-6* | 4A | 180729886 | 180730686 | 326 | GTTTGTGCGTGCGTTTGTCTC | TTGTCTCCCTATCTATACGCCACT | InDel |
| *4Aindel-7* | 4A | 236872635 | 236873435 | 180 | GGTCGGTCTCCGATTCAAAAC | TAGCATAGAGGGGGAACGGG | InDel |
| *4Aindel-8* | 4A | 291906078 | 291906878 | 263 | GTTCCGGCACCATACACTCA | CTCACACATGCAGGTCCACT | InDel |
| *4Aindel-9* | 4A | 557232206 | 557233006 | 285 | TGTTGGTGAAGATGACAGAGGT | AAACCGAGGGAAATACTTACGC | InDel |
| *ms4B-1* | 4B | 1124447 | 1125904 | 216 | GCGCCCGTGCTATATGTGA | TGGACGACAAGGAGATTACCA | SSR |
| *4Bindel-1* | 4B | 404232679 | 404233479 | 349 | TGCAATGACGTGGATGACCA | CCTGTCAGGATCGACAAGGC | InDel |
| *4Bindel-2* | 4B | 591698945 | 591699745 | 309 | CATGGTGGACACACCTAGTGAA | TCATCCGGACTGTTCTCCTCT | InDel |
| *ms5A-1* | 5A | 469993360 | 469994370 | 270 | AGCAAAGGAAGTGTGGGGC | ACGGCTAAGAGGCGATCAAC | SSR |
| *ms5A-2* | 5A | 470025795 | 470026804 | 391 | ATGAGCAATAGCGTGAGCCT | CGACAAGTCACTGTCTGCAC | SSR |
| *ms5A-7* | 5A | 470039038 | 470040047 | 351 | TGGTCCCAAAGCTCTTGCTG | CAGCCTTCCCACTGTTTTGG | SSR |
| *ms5A-3* | 5A | 470150021 | 470151032 | 355 | TGGTTACATCCCCTTGGAGC | TGGGAAATCGTTTGATGTGTTGT | SSR |
| *ms5A-4* | 5A | 470245668 | 470246715 | 145 | GGGAGACTTAGAGAGGTGGC | GATGCTTCTGATGTTGGGGGA | SSR |
| *ms5A-5* | 5A | 470332881 | 470333900 | 595 | TGAGGAAGCCCATTGTTGGT | GTCGTGGTGTAGTGGCTCTG | SSR |
| *ms5A-6* | 5A | 470394073 | 470395086 | 475 | TCCATACCAGACCAGTTGGC | ATCGCCCTGTGAGAGTGTCG | SSR |
| *ms5A-8* | 5A | 474450551 | 474451564 | 430 | CAAACAAGAGCAAACGGGCA | GGTGCTAAGGGGACAAGGAC | SSR |
| *ms5A-9* | 5A | 474497036 | 474498053 | 369 | AGATGTACTCGACGCCCAAC | CGGAAATGGTAGGAACGGCT | SSR |
| *ms5A-10* | 5A | 474729231 | 474730245 | 511 | CCCGATGCGAGTGGTTCT | ACCTCCAAGGAGCTGGTCT | SSR |
| *ms5A-11* | 5A | 474809490 | 474810500 | 316 | CCCGCACACGACTAACAGAT | GCCTAATGGGCCAAGAGAGG | SSR |
| *ms5A-12* | 5A | 474871798 | 474872813 | 197 | AGAGTAACGGTGCTTGGATCGGT | TCAAGGTGCATTAATGTTGGGGAA | SSR |
| *5Aindel-1* | 5A | 562864901 | 562865701 | 284 | GATAGTGGTGCAGGCGAAAAG | GCATGTTCCCGTATAGCCCT | InDel |
| *ms5A-14* | 5A | 607797407 | 607798418 | 515 | GCTTTGAGAGGCACGGTACT | AATCTGAGAAAAGCCCGCGA | SSR |
| *ms5A-13* | 5A | 607811064 | 607812073 | 457 | CACTGCCCAGATAGCAGTCG | GAGACGAGTTGTCCAGCCTT | SSR |
| *5Aindel-2* | 5A | 609824426 | 609825226 | 179 | ACCAGACTGAAACACCACGA | CGCCACTTCAGAACCCTTGT | InDel |
| *5Aindel-3* | 5A | 705494715 | 705495515 | 309 | CCGCACCCTCTGTTAATGGC | TATACTTACGGGTGTGGCCG | InDel |
| *5Bindel-2* | 5B | 527164603 | 527165403 | 347 | TCCCCTAACCGACTTCCTTC | TCGACTTTAGATGTATTTACGTGTC | InDel |
| *5Bindel-1* | 5B | 664470704 | 664471504 | 232 | AGTTCTCTTCGCCGCCTATG | TGCGCCTTAAAACAATGGGC | InDel |
| *5Bindel-3* | 5B | 685038480 | 685039280 | 284 | TTCGCTCCGGTTAGTGGTTTA | GCACTTGCCTCGAAGAACCA | InDel |
| *5Dindel-2* | 5D | 47080989 | 47081789 | 294 | CTTCACCAAACAACCCAAGGT | CCCCAATCATGATCATTAGCACA | InDel |
| *5Dindel-5* | 5D | 49956211 | 49957011 | 246 | CCGGCTCATAAGAGTGTCCG | GGCGACCATGTTTTCCAACG | InDel |
| *5Dindel-6* | 5D | 53842984 | 53843784 | 340 | TTCGATCCCTCTCAGCTGTC | TACATAAGCCACGAAGCAGCA | InDel |
| *5Dindel-7* | 5D | 54837575 | 54838375 | 385 | TGGAGGGACTTGTTTGCGATT | TGCCACCACGTTTCCACA | InDel |
| *5Dindel-1* | 5D | 58650338 | 58651138 | 321 | GCTAACGCTTCCGCTTTCG | TGCATGTCTGTCGGTGGAAC | InDel |
| *5Dindel-8* | 5D | 70977855 | 70978655 | 195 | GCATAAAACGCGACCTCCCC | AACCCACGCACACACACAA | InDel |
| *5Dindel-9* | 5D | 73391497 | 73392297 | 276 | ATCAGTTTCCACCCATCCCATTT | CCGTCGCACAGGGGTTAGA | InDel |
| *5Dindel-10* | 5D | 79220144 | 79220944 | 400 | GCGTAAATGAAGCCCTTGTGTTC | GCCTCAACTACATTAACGGCATA | InDel |
| *5Dindel-11* | 5D | 109745671 | 109746471 | 238 | ACCGTCTGGAAATGCTGCGTA | ATAGAGCCAGCACGATGTGAA | InDel |
| *5Dindel-12* | 5D | 142201123 | 142201923 | 262 | CATCCCAACAAACGCACCATA | AAACCACCAGTTCTTCCACGA | InDel |
| *ms5D-1* | 5D | 149207004 | 149208015 | 198 | GAGGTAGGGGATGACTGGCA | AGCATTTGAGGGCTGTTGGA | SSR |
| *ms5D-2* | 5D | 149340010 | 149341021 | 544 | TGGAAACGAAGACGGTGGCATC | TTCCAAAAAGTGGCATTGAGCAG | SSR |
| *ms5D-15* | 5D | 149362128 | 149363141 | 74 | ACCCGAGTGTCTAAGCGTCA | ACTACCGGCACATCTTCGAG | SSR |
| *ms5D-16* | 5D | 149478435 | 149479448 | 512 | CATGCTAATCTATGCACCCACAA | TCCGGAACACTTGACTGGC | SSR |
| *ms5D-3* | 5D | 149499799 | 149500814 | 482 | GTCGTGTCTGGGGCA | GAGCCATGATTAAGGGGGTTA | SSR |
| *ms5D-17* | 5D | 149558845 | 149559868 | 138 | GGTGATAGCAGAGGCGATCC | GGCCTCGAGTGTTTCTCCAA | SSR |
| *ms5D-4* | 5D | 149672013 | 149673026 | 689 | TTGAGGGCCGTCATGTATCG | TGTGGCACGTATTCAACCCA | SSR |
| *ms5D-18* | 5D | 149691104 | 149692131 | 292 | AGACCGTTCCGGCCTATACT | ACGTATCAGAGTGCAAGGAGC | SSR |
| *ms5D-5* | 5D | 149759908 | 149760917 | 399 | ATGCGGAAAACTCCCTGTCC | TTCGACCATGCTCACATACGTT | SSR |
| *ms5D-19* | 5D | 149788939 | 149789950 | 409 | GTGGTCGATGAGTCGTTGGAA | ACAGTCTGAGGAAACGGAGATG | SSR |
| *ms5D-6* | 5D | 149935588 | 149936603 | 353 | TGTATTGGCCCGTGCTAGTC | GTTGATGGGGAAGAGGGTCG | SSR |
| *ms5D-7* | 5D | 150034372 | 150035383 | 435 | GCTCGTTTCCAAACCACACC | GTGGGAAAGAGGGTGAGAGC | SSR |
| *ms5D-8* | 5D | 150229721 | 150230751 | 381 | GATCGATTGTATGCCCTGTGTGAA | ATCCTACAGTGGCCAGCTCC | SSR |
| *ms5D-9* | 5D | 150334715 | 150335741 | 449 | GGCATTTTTCTTCTTATTTAGGC | AAGGGAAACAAGCACTCCAT | SSR |
| *ms5D-10* | 5D | 150444535 | 150445546 | 445 | AGTTGCCAAGGGATGGTCC | GTTCGTATTCGTGGCTTATAGCG | SSR |
| *ms5D-11* | 5D | 150580461 | 150581478 | 525 | TCATCGCTTTTGTCTAGCAACTTT | CACAGTGGGACAATGCGACC | SSR |
| *ms5D-12* | 5D | 150698567 | 150699578 | 272 | CCAGGCCCATGGAATCTCA | CTGACCACGAGGGTCTATTTT | SSR |
| *ms5D-13* | 5D | 150782391 | 150783405 | 457 | TGCACACGGTTGACTGTTCT | AAAGCATGCCCTTTTTAGTTCTCA | SSR |
| *ms5D-14* | 5D | 150892141 | 150893158 | 210 | AGTGTTGCCTCCCAACCTTT | TCTGCGAAGGGCCTATCTTT | SSR |
| *5Dindel-4* | 5D | 153706503 | 153707303 | 217 | TCGGTGTTTTCCACAGTGCG | GAGTTGCCTCTCTACGAGCTG | InDel |
| *5Dindel-13* | 5D | 171106387 | 171107187 | 382 | TTGCAGGATGGTAGGCACAC | GTGTCACCCTTCTGCTGTCA | InDel |
| *5Dindel-14* | 5D | 181765694 | 181766494 | 362 | ATTCTCAGTGTTTCCAAATGGC | TAAGCGCCAGGTGAGTTTG | InDel |
| *6Bindel-1* | 6B | 4904271 | 4905071 | 308 | ACAGACACAAATACACATCAAGAAA | TAGTGGTAGGGAGAACCAGC | InDel |
| *6Bindel-2* | 6B | 20165559 | 20166359 | 334 | CCCTCCAGGCTACTGATCAAC | AGGGAAAAGCAAACACGAGAAA | InDel |
| *6Bindel-3* | 6B | 21193702 | 21194502 | 174 | GCGAAAAATGACCCATCAAGCA | CAGGGACTGCGGGTTGATTT | InDel |
| *6Bindel-5* | 6B | 130023497 | 130024297 | 124 | CTTGATTTTCTAGGCTTGTCAGAGG | CTAAAGAATGGGCCAAACGCC | InDel |
| *6Bindel-6* | 6B | 153911572 | 153912372 | 195 | CCAAATGTGCCCACAAGTCA | GAAGACGATAGAGGAGGCTGC | InDel |
| *6Bindel-7* | 6B | 467152732 | 467153532 | 159 | GGAAAAGTGGTTTTGTGGCCC | CGGAGACGTAAAGAGGAGGC | InDel |
| *6Bindel-8* | 6B | 689033683 | 689034483 | 317 | GGTGAGTTTGGGTATGTCCCT | GTGTTTCGCTCCTCTAGCCA | InDel |
| *7Aindel-1* | 7A | 14194313 | 14195113 | 292 | GGTAGGGGAGGCGAGTACA | TAGGGAGGGGACAGCTTTTCT | InDel |
| *7Aindel-2* | 7A | 205231902 | 205232702 | 175 | TACTATGGTGGAAGGGGGCA | GGGGAATCCTACTCCCGGTG | InDel |
| *7Aindel-3* | 7A | 224972744 | 224973544 | 186 | CCTGGCTGAACTGTGTTGTG | TGGATCAGGGGGACAAAAGC | InDel |
| *7Aindel-4* | 7A | 262089724 | 262090524 | 278 | CGCAGTCTACTTGGTTCGGA | GGCCTCTCGCCTAGTGTTTT | InDel |
| *ms7A-2* | 7A | 535955626 | 535956637 | 368 | TACCCCAGCAGCTCCATCTA | TGCACTCCTCACCAAGCAAA | SSR |
| *ms7A-3* | 7A | 536470892 | 536471903 | 547 | TAAAAGCGCATGGCAACCTC | ATACGGACATGCAGGCATCA | SSR |
| *ms7A-4* | 7A | 537062098 | 537063115 | 331 | AGCCCGTTGAGAACCAAGTC | CTTCACCACTGGCGACTACC | SSR |
| *ms7A-5* | 7A | 537730229 | 537731246 | 408 | AGAAACAGGCTTCGTTGGCA | CTCCCGAACCCTCACCCT | SSR |
| *ms7A-1* | 7A | 537948608 | 537950035 | 506 | TATTTTTACAATTCACAGCCAC | GCATCGGTATGTTTTCGGAT | SSR |
| *ms7A-6* | 7A | 538312851 | 538313864 | 296 | CGTCGCGTGCTTTTGTTACT | GGTGTAAGTTGACGCGTTGG | SSR |
| *SSR7A-7* | 7A | 538778137 | 538779150 | 254 | CATCCGGCCAGAAACACAAA | CCCTCCGTACAGCCTCTAAAC | SSR |
| *ms7A-8* | 7A | 539307541 | 539308551 | 187 | CGGACTCCGTTTGCAAATTC | TCGCCAATCTTTTTGAGGGG | SSR |
| *ms7A-9* | 7A | 539944694 | 539945704 | 148 | CTGCCCACCTTCATCCATGT | GTGACGTCTGGGGCATGTAA | SSR |
| *7Bindel-1* | 7B | 436767861 | 436768661 | 334 | TCCTAGGCCATCCTAGGGTTT | GGGGAGGAAGACGACGGA | InDel |
| *7Bindel-2* | 7B | 723134984 | 723135784 | 276 | ACGGTATTTCCATCTGCCCTG | AATTCGCATATACTCCCTCTGCAA | InDel |
| *7Dindel-1* | 7D | 191143699 | 191144499 | 240 | TAGCACATAGCCGAAAATGGC | AGAACCACGAGATCCCATCTG | InDel |
| *7Dindel-2* | 7D | 205619108 | 205619908 | 310+6 | GGCACAGAGGCACAACTAGC | CATTGCAGGATGGCAGGAAC | InDel |

Chr., chromosome; Size, PCR amplification fragment size.
